# Aggregation-Induced Synthesis (AIS): Asymmetric Synthesis via Chiral Aggregates

**DOI:** 10.34133/2022/9865108

**Published:** 2022-08-11

**Authors:** Hossein Rouh, Yao Tang, Ting Xu, Qingkai Yuan, Sai Zhang, Jia-Yin Wang, Shengzhou Jin, Yu Wang, Junyi Pan, Hannah L. Wood, James D. McDonald, Guigen Li

**Affiliations:** ^1^Department of Chemistry and Biochemistry, Texas Tech University, Lubbock, Texas 79409-1061, USA; ^2^Institute of Chemistry and BioMedical Sciences, School of Chemistry and Chemical Engineering, Nanjing University, Nanjing 210093, China

## Abstract

A new chiral aggregate-based tool for asymmetric synthesis has been developed by taking advantage of chiral aggregates of GAP (Group-Assisted Purification) reagents, *N*-phosphonyl imines. This tool was proven to be successful in the asymmetric GAP synthesis of functionalized 2,3-dihydrobenzofurans by reacting salicyl *N*-phosphonyl imines with dialkyl bromomalonates in various cosolvent systems. The chiral induction can be controlled by differentiating between two asymmetric directions simply by changing the ratios of cosolvents which are commonly adopted in AIE (aggregation-induced emission) systems. The formation of chiral aggregates was witnessed by a new analytical tool—aggregation-induced polarization (AIP). The present synthetic method will be broadly extended for general organic synthesis, particularly, for asymmetric synthesis and asymmetric catalysis in the future.

## 1. Introduction

The study of chirality and the behavior of micro- and macrochiral targets has been amongst the most important and active topics in science because technologically advanced materials heavily depend on chirality [1–9]. Increasing numbers of drugs and their chemical building blocks contain chiral subunits in their structures because drug actions require structural matching/docking so as to enhance potency and selectivity toward receptors and other targets on the surface and inside of cells [2–7, 10–13]. Structural design of chiral drugs has been playing crucial roles in reducing or avoiding severe side effects during biomedical treatments [11]. Asymmetric synthesis has thus been on the rise for half a century so as to meet challenging requirements by pharmaceutical and medical applications [12–17], as well as by nano- and photoelectronic materials [18–21]. So far, there have been four chemical strategies for controlling molecular chirality by the use of chiral catalysts, chiral auxiliaries, chiral reagents, and chiral solvents. To the best of our knowledge, there are no other asymmetric controllers beyond the aforementioned methods documented in the collective of scientific literature.

In the past several years, our lab has established the Group-Assisted Purification (GAP) chemistry and technology by taking advantage of new *N*-phosphonyl and *N*-phosphinyl imine reagents and their usages for asymmetric synthesis [22–31]. The existence of GAP functions in chiral imine starting materials can avoid the formation of oily and sticky products and instead result in crystalline solids, including chiral or achiral aggregates. Therefore, GAP functions enable isolation and purification simply by washing with common solvents or cosolvents, thereby avoiding traditional column chromatography and recrystallization. GAP chemistry provides an asymmetric tool belonging to the category of both chiral auxiliaries and chiral reagents in asymmetric synthesis since the amino functionality of products is derived from GAP reagents. When GAP chemistry is utilized for peptide synthesis, shortcomings of both solid- and solution-phase syntheses can be avoided. Furthermore, we found that GAP groups can substantially increase chemical yields for peptide synthesis which is defined as Group-Assisted Synthesis (GASyn) [32] or aggregation-induced synthesis (AIS). In fact, GASyn or AIS chemistry often showed nearly quantitative yield for each step of the polypeptide synthesis. This indicates that GAP aggregation would serve as a new synthetic tool between traditional homogeneous and heterogeneous protocols. The corresponding soluble soft matters would also result in higher effectiveness for synthetic reactions. It is noteworthy that GAP chemistry has enabled the Fmoc group, the most welcome protection group in peptide synthesis, to be of use in the solution phase for the first time [33, 34]. GAP chemistry would be the only chemical concept that combines the following four aspects into one: reagents, reaction, separation, and purification. This takes into account both reactants and products while considering their chemical and physical factors in regard to reactivity, selectivity, stability, and solubility. For asymmetric synthesis and catalysis, GAP chemistry should be able to control diastereo-, enantio-, and chemoselectivity and to recover and recycle catalysts for reusage [34, 35]. This is indeed the third environmentally friendly method for recycling catalysts in addition to polymer- and organic salt-based tools in which various shortcomings exist in their applications. The GAP catalysts and chiral aggregates formed during catalysis processes make it possible to develop a new catalysis manner between traditional homogeneous and heterogeneous protocols.

## 2. Results

### 2.1. Hypothesis and Design Rationale

**Scheme 1:**
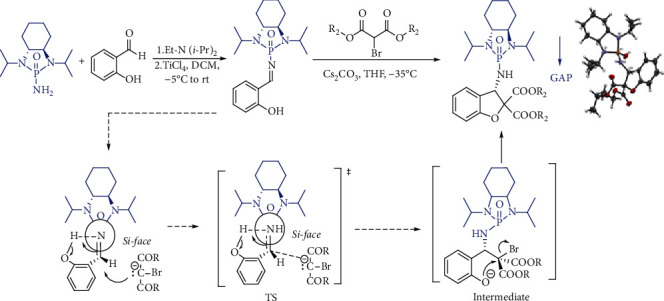
Asymmetric GAP synthesis of 2,3-dihydrobenzofuran.

Very recently, we reported the asymmetric GAP synthesis of functionalized 2,3-dihydrobenzofurans with biological screening potentials via domino-annulation between salicyl *N*-phosphonyl imines and dialkyl bromomalonates (Scheme 1) [36]. A less-nucleophilic cesium carbonate base was employed to afford products in good to excellent yields and diastereoselectivity. 2,3-Dihydrobenzofuran products were separated/purified simply by washing with hexanes to bypass conventional separation methods. The reaction occurred via nucleophilic addition from the *Si* face on the chiral GAP imines to form C-C bonds subsequently followed by intramolecular electrophilic S*_N_*2 reaction to form a C-O bond. Hydrogen bonding would be formed between OH and a lone-pair of electrons of C=N in the chiral GAP imine prior to S*_N_*2 electrophilic substitution.

The GAP function with a polar moiety in the transition states and intermediates during the reaction process (Scheme 1) is anticipated to provide the foundation of aggregates formed in intermolecular manners. This inspired us to envision the possible formation of chiral aggregates indicating different asymmetric controls. Preferably, these chiral aggregates would drive the asymmetric reactions in opposite directions while keeping the chirality of starting materials unchanged. This is particularly important for industrial-scale production in the future because in existing asymmetric protocols, the opposite chiral control has to be achieved by changing chirality in auxiliaries, catalysts, reagents, or solvents. Pleasantly, our hypothesis and design rationale have now been proven to be feasible by performing a series of asymmetric experiments. Herein, we would like to present our preliminary results of this study.

### 2.2. Synthesis and Stereoselectivity Determination

**Figure 1:**
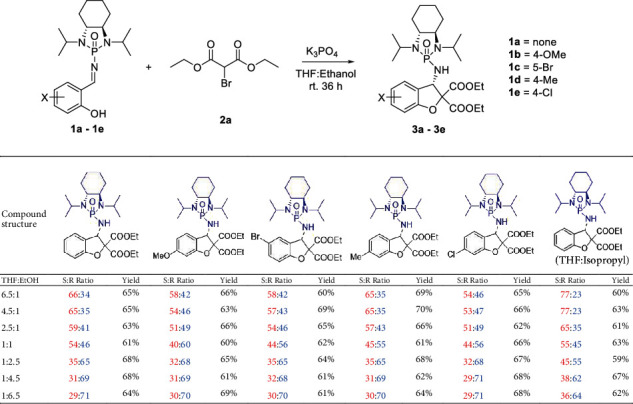
Results of chiral aggregate-induced synthesis.

**Figure 2:**
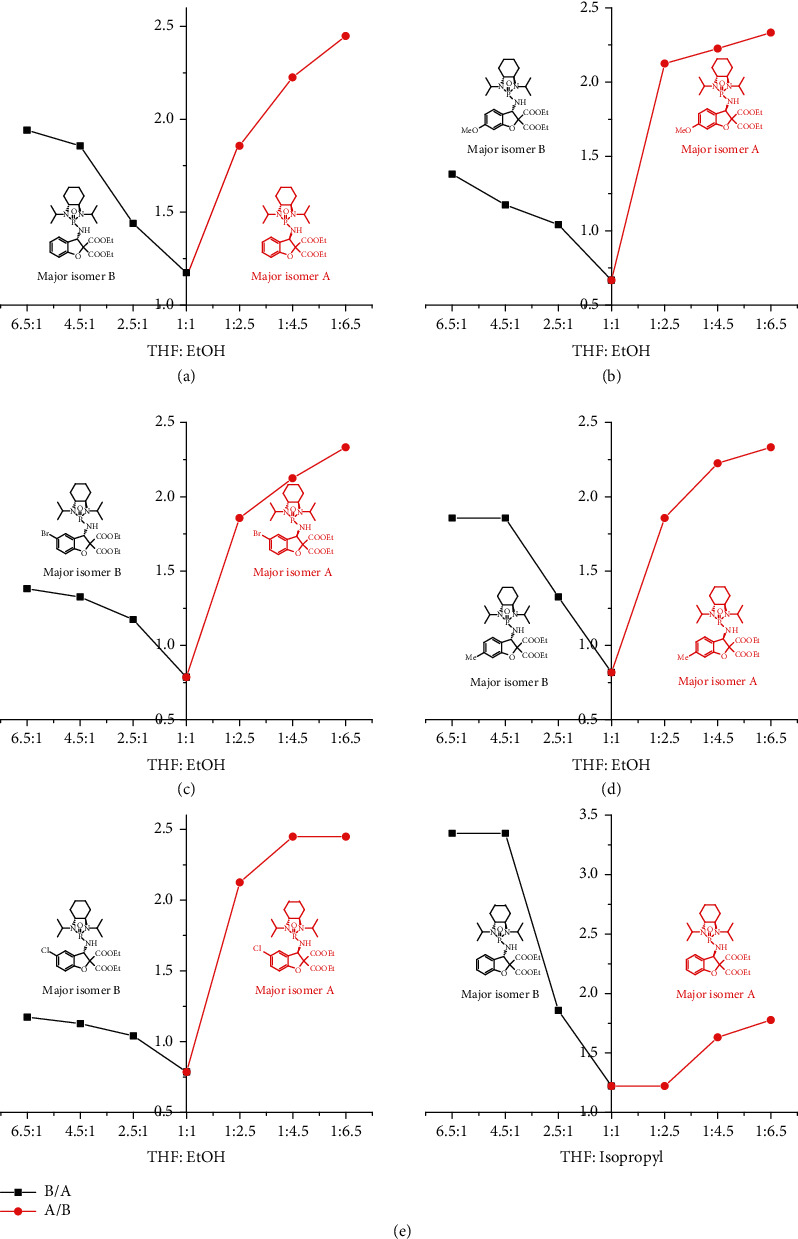
Curve description of chiral aggregate-induced asymmetric synthesis.

At the onset, our investigation was based on our previous results on the synthesis of 2,3-dihydrobenzofurane via Group-Assisted Purification chemistry for which a suitable reaction condition must be selected. Salicyl *N*-phosphonyl imine 1a was treated with diethyl bromomalonate 2a (2.0 equiv.) in the presence of potassium phosphate (2.0 equiv.) at room temperature. Typical aggregation solvents of THF : H_2_O in three ratios of (3 : 1, 1 : 1, and 1 : 3) were employed for this reaction. Surprisingly, the product could not be formed in the above solutions. Changing the inorganic base to potassium carbonate did not result in any product either. We then decided to use another polar protonic solvent, ethanol, to replace water for the aggregation system. Although the combination of THF/EtOH/K_2_CO_3_ did not show any success, 2,3-dihydrobenzofuran was formed when the reaction was performed in THF and ethanol (*v*/*v* = 1 : 1) with potassium phosphate as the deprotonation base. The product appeared as a diastereomeric mixture with a ratio of 45(S) : 55(R) based on crude ^31^P NMR analysis. Subsequently, the relationship between solvent ratios and diastereoselectivity was investigated by setting the ratios of ethanol and THF toward two directions on the *X*-horizontal coordinate correlating to diastereoselectivity on the *Y*-vertical ordinate. The ratio of *v*/*v* = 1 : 1 was set in the middle, as the reference on the *X*-horizontal coordinate. The reaction was performed in seven sets of cosolvents of THF/ethanol: 6.5 : 1, 4.5 : 1, 2.5 : 1, 1 : 1, 1 : 2.5, 1 : 4.5, and 1 : 6.5 (Figures 1 and 2).

After the complete consumption of starting materials of the reaction, the measurements of chemical yields were also based on crude ^31^P NMR analysis by using triphenylphosphine as the internal standard in which the ^31^P NMR peak appears at -5 ppm. In all cases, for all aromatic substrates with either neutral, electron-withdrawing, or electron-donating groups, the estimated yields arrange from 60% to 70%, which is within a similar range of isolated yields as our previous synthesis.

As demonstrated in Figures 1 and 2, increasing the amount of polar solvent of ethanol led to the formation of the diastereomer with an R configuration arising as the major isomer. In contrast, increasing the amount of THF resulted in the diastereomer with an S configuration as the major isomer. For case 1, without any substitution on the phenyl ring, a reversed jump of diastereoselectivity from 54 : 46 (S : R, *v*/*v* = 1/1) to 35 : 56 (v − EtOH/v − THF = 2.5/1) occurred from the S to R major isomer, while the increased diastereoselectivity from 54 : 46 (S : R, *v*/*v* = 1/1) to 59 : 41 (S : R, v − THF/v − EtOH = 2.5/1) was observed with the S major isomer. The trend of R isomer predominant formation was maintained up to 71 : 29 (R : S) when the ratios of v-EtOH/v-THF reached 6.5/1. Similarly, the trend of the S isomer predominant formation was maintained up to 66 : 34 (R : S) when the ratios of v-THF/v-EtOH reached 6.5/1, albeit the diastereoselectivity is not as high as that of the R isomeric counterpart. For case 2, with a strong electron-donating attachment (–OMe) on position 3 of the phenyl ring, a large jump of diastereoselectivity from 40 : 60 (S : R, *v*/*v* = 1/1) to 68 : 32 (R : S, v − EtOH/v − THF = 2.5/1) occurred from the S to R major isomer, while the increased diastereoselectivity from the same ratio of 40 : 60 to 51 : 49 (R : S, v − THF/v − EtOH = 2.5/1) was observed with the S major isomer. Interestingly, the same observation with a modest electron-withdrawing group (-Cl) on position 3 of the phenyl ring (case 5) was made with the same jump of diastereoselectivity from 44 : 56 (S : R, *v*/*v* = 1/1) to 68 : 32 (R : S, v − EtOH/v − THF = 2.5/1) which occurred from the S to R major isomer, while the increased diastereoselectivity from the same ratio of 40 : 56 to 51 : 49 (S : R, v − THF/v − EtOH = 2.5/1) was observed with the S major isomer. This observation indicates that a polar group on position 3 of the phenyl ring of the starting materials would favor the formation of chiral aggregates for more efficient asymmetric control.

Cases 3 and 4 have relatively weak electron-donating attachments (Me and Br) on its phenyl rings on positions 3 and 4, respectively. These two were found to have a similar relationship of diastereoselectivity with cosolvent ratios to that of case 1 in which there is no additional substitution on its aromatic ring. The diastereoselectivity enhancement of the S isomer formation for these two cases is also very close when cosolvent ratios of v-THF/v-EtOH were increased from 1/1 to 6.5/1.

In all the above cases 1-5, the control of R isomers predominates that of S isomers in consistently increasing ratios of v-EtOH/v-THF (red curves in Figures 2(a)–2(d)). However, when isopropanol (iPrOH) was utilized to replace ethanol for case 1, this situation was reversed. In this case, the S isomer's control predominated R isomers with consistently increased ratios of v-THF/v-iPrOH (Figure 2(e)). A big jump occurred when v-THF/v-iPrOH was increased from 2.5/1 to 4.5/1 providing the major S isomeric product, diethyl (3S)-3-((1,3-diisopropyl-2-oxidooctahydrobenzo[d] [1,3,2] diazaphosphol-2-yl)amino)-5-methylbenzofuran-2,2(3H)-dicarboxylate (3d). Furthermore, the highest diastereoselectivity of S/R = 77 : 23 was achieved under this condition.

### 2.3. Aggregation Determination

**Figure 3:**
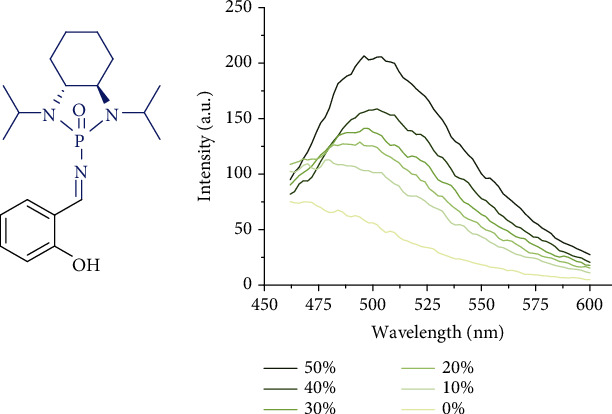
PL spectra of salicyl N-phosphonyl imine in THF/EtOH cosolvents with different water fractions (*f*_w_); *c* = 0.1 mM; *λ*ex = 400 nm.

Since THF and EtOH are typical cosolvents consisting of nonprotonic/less polar and protonic/more polar components for aggregation-induced emission (AIE) [18, 37, 38], we measured AIE of chiral *N*-phosphonyl imine (1a) in these solvents to support the formation of aggregates. After carefully adjusting the irradiation power (900 V) and wavelength (*λ*ex = 400 nm), as shown in Figure 3, this compound displayed an obvious AIE indicating an efficient formation of aggregates in THF/EtOH systems. The emission maxima of 1a were gradually increased as the water fractions (*f*_w_) were changed from 10% to 50%.

**Figure 4:**
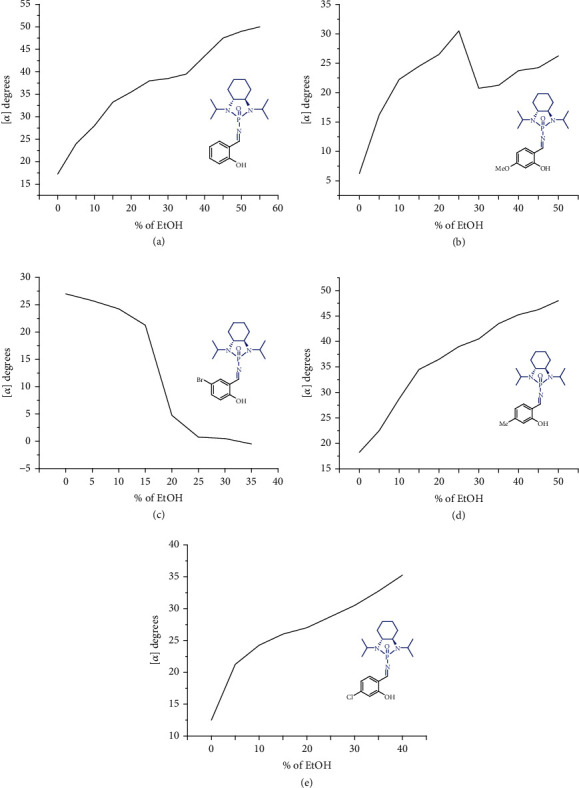
Aggregation-induced polarization of GAP imines in THF/EtOH cosolvents; *c* = 4 mg/mL.

The above evidence of forming chiral aggregates would make current AIS feasible and enable the correlation of aggregation with aggregation-induced polarization (AIP) (Figure 4). The AIP measurement was started on compound 1 under the present asymmetric synthesis system. A Rudolph polarimeter (Rudolph Research Analytical APIV/2W) was utilized to acquire optical rotation data at around room temperature with a sodium lamp as the light source (wavelength = 589 nm). Measurements were performed in a vessel of 2 mL with a consistent concentration (*c* = 4 mg/mL) in THF and EtOH cosolvents. The average data of three measurements for each sample were utilized in plotting the relationship curves.

In this measurement, the ethanol fraction (*f*_EtOH_) was set at a component of 5% (*v*/*v*) on the *X*-horizontal axis corresponding to a specific rotation on the *Y*-vertical coordinate. As revealed in Figure 4, under the standard aggregation cosolvents of THF and EtOH, *N*-phosphonyl imine 1a showed a consistent relationship between its optical rotation with ethanol fractions by gradually increasing EtOH in the cosolvents. The specific optical rotation was substantially enhanced from 17.3° to 49.0° when *f*_EtOH_ was increased from 0% to 50%; this trend is kept constant (Figure 4(a)). *N*-Phosphonyl imine 1b showed a similar polarization enhancement from 6.3° to 30.5° during the range of ethanol fractions (*f*_EtOH_) from 0% to 25% but drops to 20.8 ^o^ after *f*_EtOH_ reached 30%. Interestingly, the trend of polarization enhancement was resumed again during the *f*_EtOH_ range between 30% and 50% (Figure 4(b)). The optical rotation of *N*-phosphonyl imine 1c was kept dropping from 27.5° to about -0.5°. The GAP starting material 1d presented a similar phenomenon to that of 1a with a consistent enhancement of optical rotation from 18.3° to 48° when from 0% to 50% (Figure 4(d)). For GAP imine 1e, the optical rotation resumed enhancement from 12.5° to 37.8° when *f*_EtOH_ was increased from 0% to 40% (Figure 4(e)). Since the absolute configuration is usually unpredictable for individual chiral molecules, their optical rotation of aggregation would be anticipated to be complicated and require further investigation and computational study in the future.

As shown above, AIP would serve as a new tool to determine molecular aggregation. It can also result in systematic polarization enhancement and adjustment of chiral targets simply by changing cosolvents. Meanwhile, AIP and AIE-based CPL would complement each concerning generating right- or left-polarized lights for academic research and technology applications in the future; i.e., the right- or left-polarized light beams via AIP transmission are originally emitted from metal or metal filament cycle lamps (external light sources) which belong to laser beams of individual wavelengths. In contrast, the right- or left-polarized light beams generated from AIE-based CPL from emission are generated by aggregation particles themselves.

It should also be pointed out that the research on aggregates has been primarily focused on concepts pertaining to physics with nearly no documentation on chemical synthesis so far. In organic synthesis, it has been commonly believed that individual molecules of starting materials are responsible for the formation of products. This acceptance is about the behaviors of freely distributed single molecules in a reaction solution for homogeneous systems. The present work indicates a new understanding that many reactions would involve individual molecules, their dimers, trimers, etc. More importantly, they would also consist of a series of aggregates, enabling reactions to afford different outcomes. The formation of achiral and chiral aggregates during the reaction process would depend on various factors, such as concentrations, solvents, temperature, additives, and pressure. This is particularly important for asymmetric synthesis and catalysis controlled by chiral aggregates which can be formed from chiral starting materials and individual chiral catalyst molecules. The present asymmetric synthesis using chiral aggregates would be a new addition to the family of asymmetric controlling tools of using chiral auxiliaries, reagents, solvents, and catalysts. The opposite control provides a unique tool to control the chirality of organic and medicinal targets and their building blocks without changing the chirality of the above four entities, enabling this work to be a greener and more environmentally friendly chemistry. Therefore, simply changing inexpensive solvents and less or nontoxic can result in opposite isomeric products, which can avoid the tedious synthesis of chiral starting materials and minimize manpower and energy usage and reduce waste generation.

## 3. Discussion

In conclusion, we have established a new tool, aggregation-induced asymmetric synthesis (AIAS), for asymmetric synthesis of chiral products by taking advantage of chiral aggregates of GAP (Group-Assisted Purification) reagents, *N*-phosphonyl imines. The asymmetric GAP synthesis of functionalized 2,3-dihydrobenzofurans by reacting salicyl *N*-phosphonyl imines with dialkyl bromomalonates was conducted in various cosolvents of typical aggregation systems. By changing the ratios of cosolvents (THF and EtOH), the chiral products were directed toward different directions; i.e., (*R*)-isomeric products were predominantly formed over (*S*)-counterparts when amounts of EtOH were increased and vice versa. The formation of chiral aggregates was witnessed by AIE and, concurrently, by a new analytical tool—aggregation-induced polarization (AIP) of chiral starting materials in typical AIE cosolvents. The present synthetic method would be defined as aggregation-induced asymmetric synthesis, aggregation-assisted asymmetric synthesis, aggregation-induced asymmetric reaction, or aggregation-assisted asymmetric reaction. The chiral aggregate-based asymmetric strategy will be broadly extended for general organic synthesis, particularly, for asymmetric synthesis and asymmetric catalysis in the future.

## 4. Materials and Methods

Unless otherwise stated, all reactions were magnetically stirred and conducted in oven-dried glassware in anhydrous solvents under Ar, applying standard Schlenk techniques. Solvents and liquid reagents, as well as solutions of solid or liquid reagents, were added via syringes and stainless steel or polyethylene cannulas through rubber septa or through a weak Ar counterflow. Solvents were removed under reduced pressure at 40-65°C using a rotavapor. All given yields are isolated yields of chromatographic and NMR spectroscopic materials. The internal standard was performed for yield measurements as well. All commercially available chemicals were used as received without further purification. Solvents were obtained as follows: EtOH, *i*PrOH, and THF are delivered from an Innovation Technology solvent system.

The ^1^H and ^13^C NMR spectra were recorded in CDCl_3_ or DMSO-*d*_6_ on 400 MHz and 500 MHz instruments with TMS as the internal standard. For referencing of the ^1^H NMR spectra, the residual solvent signals (*δ* = 7.26 for CDCl_3_ and *δ* = 2.50 for DMSO-*d*_6_) were used. In the case of the ^13^C NMR spectra, the signals of solvents (*δ* = 7.16 for CDCl_3_ and *δ* = 39.52 for DMSO-*d*_6_) were used. Chemical shifts (*δ*) were reported in ppm with respect to TMS. Data are represented as follows: chemical shift, multiplicity (s = singlet, d = doublet, t = triplet, and m = multiplet), coupling constant (*J*, Hz), and integration. ^31^P NMR spectra were referenced to external H_3_PO_4_ (0.00 ppm). Fluorescence data were collected using the Cary Eclipse Fluorescence Spectrophotometer and Eclipse ADL program. Measurements were performed with diluted samples with 0.1 mM concentrations at 400 nm maximum excitation wavelength under electric power at 900 V. Optical rotations were measured with a Rudolph Research Analytical APIV/2W polarimeter at the indicated temperature with a sodium lamp. Measurements were performed at 2 mL with the concentration unit of g/100 mL in the corresponding solvents.

## Data Availability

All data are available in the manuscript or supplementary materials.

## Supplementary Materials

Supplementary Materials General synthesis of salicyl N-phosphonyl imines (1a-1d) and 2,3-dihydrobenzofuran (3a-3e) and their analytical data, ^1^H NMR spectra, ^13^C NMR spectra, and ^31^P NMR spectra. ^31^P NMR spectra for diastereoselectivity determination.
