# Supplementary material for: Aggregation-Induced Synthesis (AIS): Asymmetric Synthesis via Chiral Aggregates
Source: Research (Wash D C). 2022 Aug 11;2022:9865108. doi: 10.34133/2022/9865108 (PMC9394053; doi:10.34133/2022/9865108)

Supplementary Materials

Aggregation-Induced Synthesis (*AIS*): Asymmetric Synthesis via Chiral Aggregates

Hossein Rouh,[1,†] Yao Tang,[1,†] Ting Xu,[2,†] Qingkai Yuan,[1] Sai Zhang,[1] Jia-Yin Wang,[2] Shengzhou Jin,[2] Yu Wang,[2] Junyi Pan,[2] Hannah Wood,[1,#] James D. McDonald[1,#] and Guigen Li[1,2,*]

[1]*Department of Chemistry and Biochemistry, Texas Tech University, Lubbock, Texas 79409-1061, USA.*
[2]*Institute of Chemistry and BioMedical Sciences, School of Chemistry and Chemical Engineering, Nanjing University, Nanjing, 210093, China*

EXPERIMENTAL SECTION

General Aspects.

All commercially available chemicals were used as received without further purification. Solvents were obtained as follows: ether, dichloromethane, tetrahydrofuran, and toluene were delivered from an Innovation Technology solvent system. All reactions were carried out in a flame-dried flask under nitrogen gas. The 1H and 13C NMR spectra were recorded in CDCl3 on a 400 MHz instrument with TMS as the internal standard. Chemical shifts (δ) were reported in ppm with respect to TMS. Data are represented as follows: chemical shift, mutiplicity (s = singlet, d = doublet, t = triplet, m = multiplet), coupling constant (J, Hz), and integration. 31P NMR spectra were referenced to external H_3PO_4 (0.00 ppm). Shifts in 19F NMR spectra were reported based on an external hexafluorobenzene reference. HRMS analyses were carried out using a TOF-MS instrument with an ESI source.

General synthesis of salicyl N-phosphonyl imine (1a-1d): Into an oven-dried round bottom flask, flushed and protected by argon, phosphoramide (1g, 3.85 mmol) and salicylaldehyde (5.78 mmol) were dissolved in 40 ml dry dichloromethane. After 5 minutes, the mixture was cooled to -10o C, followed by drop-wise addition of diisopropylethylamine (11.56 mmol) and TiCl4 in DCM (1M, 3.08 mmol). The reaction stirred at -10oC for 30 minutes and room temperature overnight. Next, the mixture was concentrated to 5 ml by rotary evaporation in vacuo. The resultatnt solution was passed through a pad of silica gel, eluted with Hexanes: Ethyl acetate (v/v 7:3 to 3:7) to provide salicyl N-phosphonyl imine as a yellow solid.

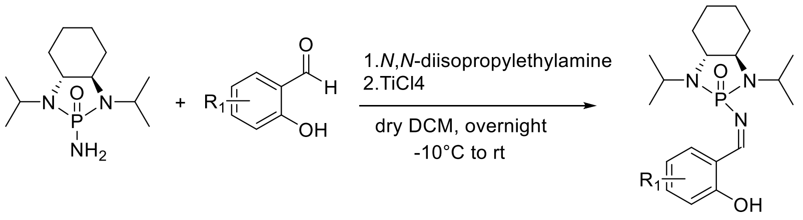

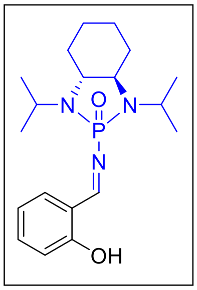

***(3aR,7aR)-2-((2-hydroxybenzylidene)amino)-1,3-diisopropyloctahydrobenzo[d][1,3,2]diazaphosphole 2-oxide* (1a).** Yellow solid, 0.981 g, 70% yield; $[\alpha]_D^{25}$ +10.6 (c 0.45, $CHCl_3$); ^{1}H NMR (400 MHz, $CDCl_3$) δ 9.09 (d, J = 29.0 Hz, 1H), 7.45 – 7.36 (m, 2H), 7.00 – 6.88 (m, 2H), 3.46 – 3.26 (m, 2H), 3.11 – 3.02 (m, 1H), 2.90 (td, J = 10.4, 3.1 Hz, 1H), 2.14 – 1.92 (m, 2H), 1.80 (d, J = 6.4 Hz, 2H), 1.40 – 1.27 (m, 3H), 1.27 – 1.14 (m, 10H), 1.09 (d, J = 6.7 Hz, 3H); ^{13}C NMR (101 MHz, $CDCl_3$) δ 175.50 (s), 175.44 (s), 162.84 (d, J = 1.9 Hz), 135.04 (s), 133.94 (s), 119.28 (s), 117.62 (s), 60.01 (d, J = 2.5 Hz), 59.92 (d, J = 2.4 Hz), 45.09 (d, J = 3.3 Hz), 44.49 (d, J = 3.6 Hz), 29.85 (d, J = 9.7 Hz), 29.57 (d, J = 9.5 Hz), 24.41 – 24.31 (m), 21.98 (d, J = 3.7 Hz), 21.21 (s), 21.19 (s), 20.85 (s), 20.38 (d, J = 1.5 Hz); ^{31}P NMR (162 MHz, $CDCl_3$) δ 21.81. ; HRMS (TOF ES+) m/z calcd for $C_{19}H_{30}N_3O_2P$ [$(M + H)^+$], 364.207; found, 364.216

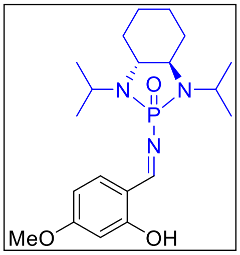

***(3aR,7aR)-2-((2-hydroxy-4-methoxybenzylidene)amino)-1,3-diisopropyloctahydrobenzo[d][1,3,2]diazaphosphole 2-oxide* (1b).**Yellow solid, 0.971 g, 64% yield; $[\alpha]_D^{25}$ -16.7 (c 0.65, $CHCl_3$); ^{1}H NMR (400 MHz, $CDCl_3$) δ 8.83 (d, J = 25.6 Hz, 1H), 7.23 (d, J = 8.7 Hz, 1H), 6.41 (dd, J = 8.7, 2.4 Hz, 1H), 6.35 (d, J = 1.8 Hz, 1H), 3.81 (s, 3H), 3.48 – 3.27 (m, 2H), 3.15 – 2.97 (m, 1H), 2.94 – 2.83 (m, 1H), 2.03 (dd, J = 7.4, 5.3 Hz, 2H), 1.80 (d, J = 4.6 Hz, 2H), 1.34 (dd, J = 11.6, 4.6 Hz, 3H), 1.26 – 1.17 (m, 10H), 1.10 (d, J = 6.7 Hz, 3H); ^{13}C NMR (101 MHz, $CDCl_3$) δ 171.91 (d, J =

4.6 Hz), 168.74 (s), 166.24 (s), 135.38 (s), 113.22 (d, J = 18.1 Hz), 108.36 (s), 101.26 (s), 60.03 (s), 59.93 (d, J = 2.6 Hz), 59.82 (s), 55.63 (s), 45.06 (d, J = 3.2 Hz), 44.47 (d, J = 3.6 Hz), 29.86 (d, J = 9.7 Hz), 29.48 (d, J = 9.6 Hz), 24.35 (dd, J = 3.0, 1.2 Hz), 21.91 (d, J = 3.8 Hz), 21.16 (d, J = 2.7 Hz), 20.86 (d, J = 1.4 Hz), 20.32 (d, J = 1.4 Hz); ^{31}P NMR (162 MHz, $CDCl_3$) 21.01 ; HRMS (TOF ES+) m/z calcd for $C_{20}H_{32}N_3O_3P$ [$(M + H)^+$], 394.218; found, 394.226

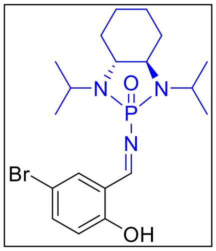

(3aR,7aR)-2-((5-bromo-2-hydroxybenzylidene)amino)-diisopropyloctahydrobenzo[d][1,3,2]diazaphosphole 2-oxide **(1c).**Yellow solid, 1.16 g, 68% yield; $[\alpha]_D^{25}$ +16 (c 0.45, $CHCl_3$); ^1H NMR (400 MHz, $CDCl_3$) δ 9.01 (d, J = 28.4 Hz, 1H), 7.52 (d, J = 2.5 Hz, 1H), 7.47 (dd, J = 8.8, 2.4 Hz, 1H), 6.88 (d, J = 8.9 Hz, 1H), 3.46 – 3.28 (m, 2H), 3.16 – 3.01 (m, 1H), 2.96 – 2.79 (m, 1H), 2.11 – 2.03 (m, 2H), 1.82 (d, J = 7.2 Hz, 2H), 1.44 – 1.30 (m, 3H), 1.27 – 1.16 (m, 10H), 1.09 (d, J = 6.7 Hz, 3H); ^{13}C NMR (101 MHz, $CDCl_3$) δ 173.70 (d, J = 6.1 Hz), 161.93 (d, J = 1.9 Hz), 137.60 (s), 135.68 (s), 120.46 (d, J = 20.0 Hz), 119.76 (s), 110.68 (d, J = 1.3 Hz), 60.03 (d, J = 6.9 Hz), 59.94 (d, J = 7.2 Hz), 45.18 (d, J = 3.2 Hz), 44.54 (d, J = 3.5 Hz), 29.78 (d, J = 9.7 Hz), 29.55 (d, J = 9.4 Hz), 24.34 (s), 21.97 (s), 21.94 (s), 21.19 (d, J = 2.8 Hz), 20.85 (d, J = 1.3 Hz), 20.45 (d, J = 1.5 Hz); ^{31}P NMR (162 MHz, $CDCl_3$) δ 21.44. ; HRMS (TOF ES+) m/z calcd for $C_{19}H_{29}BrN_3O_2P$ [$(M + H)^+$], 443.118; found, 442.127

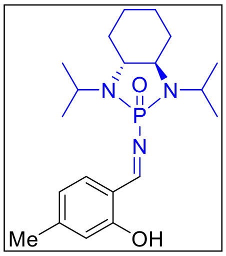

(3aR,7aR)-2-((2-hydroxy-4-methylbenzylidene)amino)-1,3-diisopropyloctahydrobenzo[d][1,3,2]diazaphosphole 2-oxide **(1d).**Yellow solid, 0.932 g, 64% yield; $[\alpha]_D^{25}$ +17 (c 0.3, $CHCl_3$); ^1H NMR (400 MHz, $CDCl_3$) δ 9.01 (d, J = 28.9 Hz, 1H), 7.26 (d, J = 8.3 Hz, 1H), 6.73 (s, 1H), 6.70 (d, J = 7.9 Hz, 1H), 3.45 – 3.23 (m, 2H), 3.09 – 2.98 (m, 1H), 2.86 (td, J = 10.2, 3.1 Hz, 1H), 2.30 (s, 3H), 2.09 – 1.98 (m, 2H), 1.77 (d, J = 6.0 Hz, 2H), 1.23 – 1.15 (m, 10H), 1.06 (d, J = 6.7 Hz, 3H); ^{13}C NMR (101 MHz, $CDCl_3$) δ 174.90 (d, J = 6.2 Hz), 163.04 (d, J = 1.9 Hz), 146.67 (s),

133.76 (s), 120.56 (s), 117.86 (d, J = 0.8 Hz), 117.01 (d, J = 20.0 Hz), 59.97 (d, J = 4.6 Hz), 59.88 (d, J = 4.5 Hz), 45.04 (d, J = 3.3 Hz), 44.44 (d, J = 3.6 Hz), 29.83 (d, J = 9.8 Hz), 29.54 (d, J = 9.4 Hz), 24.34 (dd, J = 2.0, 1.3 Hz), 22.13 (s), 21.93 (s), 21.90 (s), 21.15 (d, J = 2.8 Hz), 20.83 (d, J = 1.4 Hz), 20.32 (d, J = 1.5 Hz); ^{31}P NMR (162 MHz, $CDCl_3$) 22.01 ; HRMS (TOF ES+) m/z calcd for $C_{20}H_{32}N_3O_2P$ [(M + H)$^+$], 378.223; found, 378.231

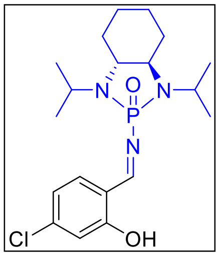

***(3aR,7aR)-2-((4-chloro-2-hydroxybenzylidene)amino)-1,3-diisopropyloctahydrobenzo[d][1,3,2]diazaphosphole 2-oxide* (1e).** Yellow solid, 1.04 g, 68% yield; $[\alpha]_D^{25}$ +8.88 (c 0.45, $CHCl_3$); ^{1}H NMR (400 MHz, $CDCl_3$) δ ^{1}H NMR (400 MHz, CHLOROFORM-D) δ 9.03 (d, J = 28.0 Hz, 1H), 7.32 (d, J = 8.3 Hz, 1H), 6.96 (d, J = 2.0 Hz, 1H), 6.87 (dd, J = 8.3, 1.9 Hz, 1H), 3.44 – 3.26 (m, 2H), 3.11 – 3.01 (m, 1H), 2.95 – 2.83 (m, 1H), 2.12 – 1.98 (m, 2H), 1.80 (d, J = 6.4 Hz, 2H), 1.42 – 1.25 (m, 3H), 1.26 – 1.12 (m, 11H), 1.09 (d, J = 6.7 Hz, 3H); ^{13}C NMR (101 MHz, $CDCl_3$) δ 174.12 (d, J = 5.9 Hz), 164.11 (d, J = 1.9 Hz), 141.26 (s), 134.71 (s), 119.85 (s), 118.10 (d, J = 0.7 Hz), 117.71 (d, J = 19.6 Hz), 60.04 (d, J = 2.2 Hz), 59.95 (d, J = 2.0 Hz), 45.12 (d, J = 3.3 Hz), 44.49 (d, J = 3.6 Hz), 29.83 (d, J = 9.7 Hz), 29.52 (d, J = 9.5 Hz), 25.34 – 23.53 (m), 22.01 (s), 21.97 (s), 21.17 (d, J = 2.8 Hz), 20.86 (d, J = 1.4 Hz), 20.39 (d, J = 1.5 Hz); ^{31}P NMR (162 MHz, $CDCl_3$) 21.33 ; HRMS (TOF ES+) m/z calcd for $C_{19}H_{29}ClN_3O_2P$ [(M + H)$^+$], 398.168; found, 398.177

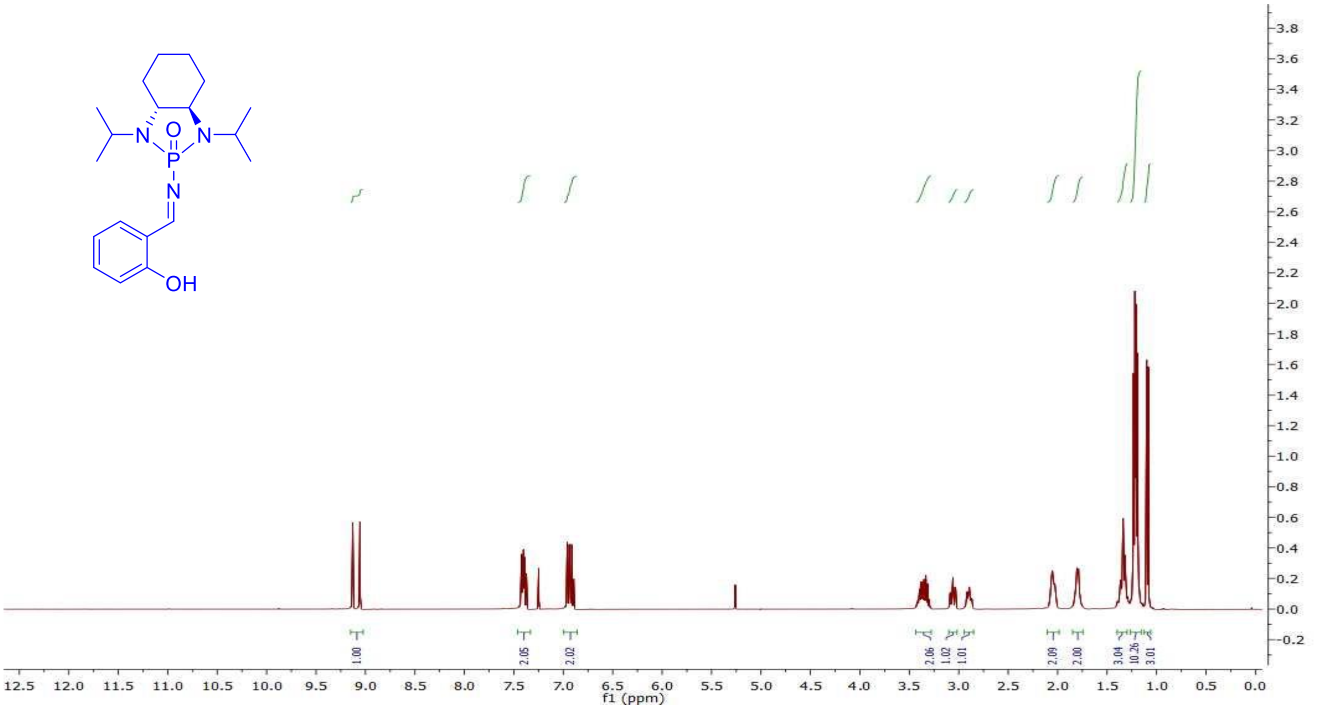

^{1}H NMR of imine **1a** ($CDCl_3$, 400 Hz)

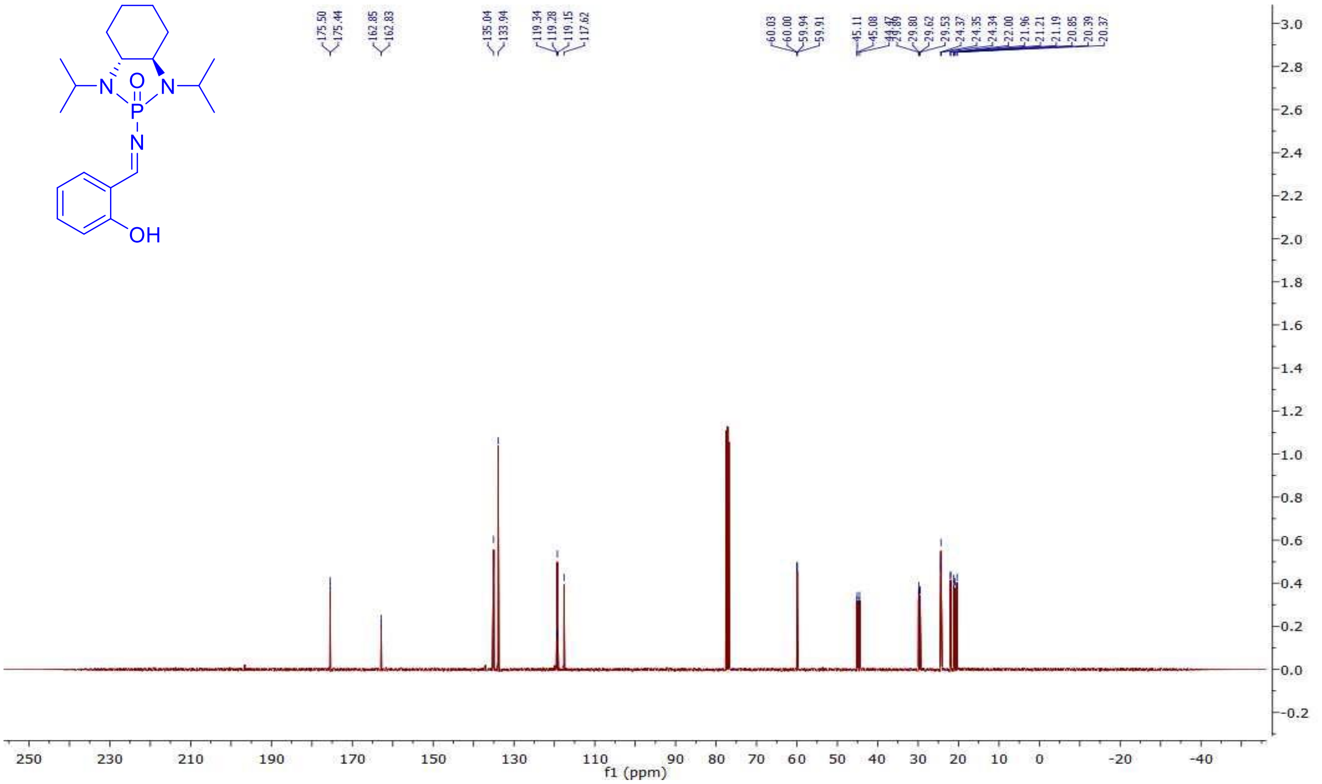

^{13}C NMR of imine **1a** ($CDCl_3$, 100 Hz)

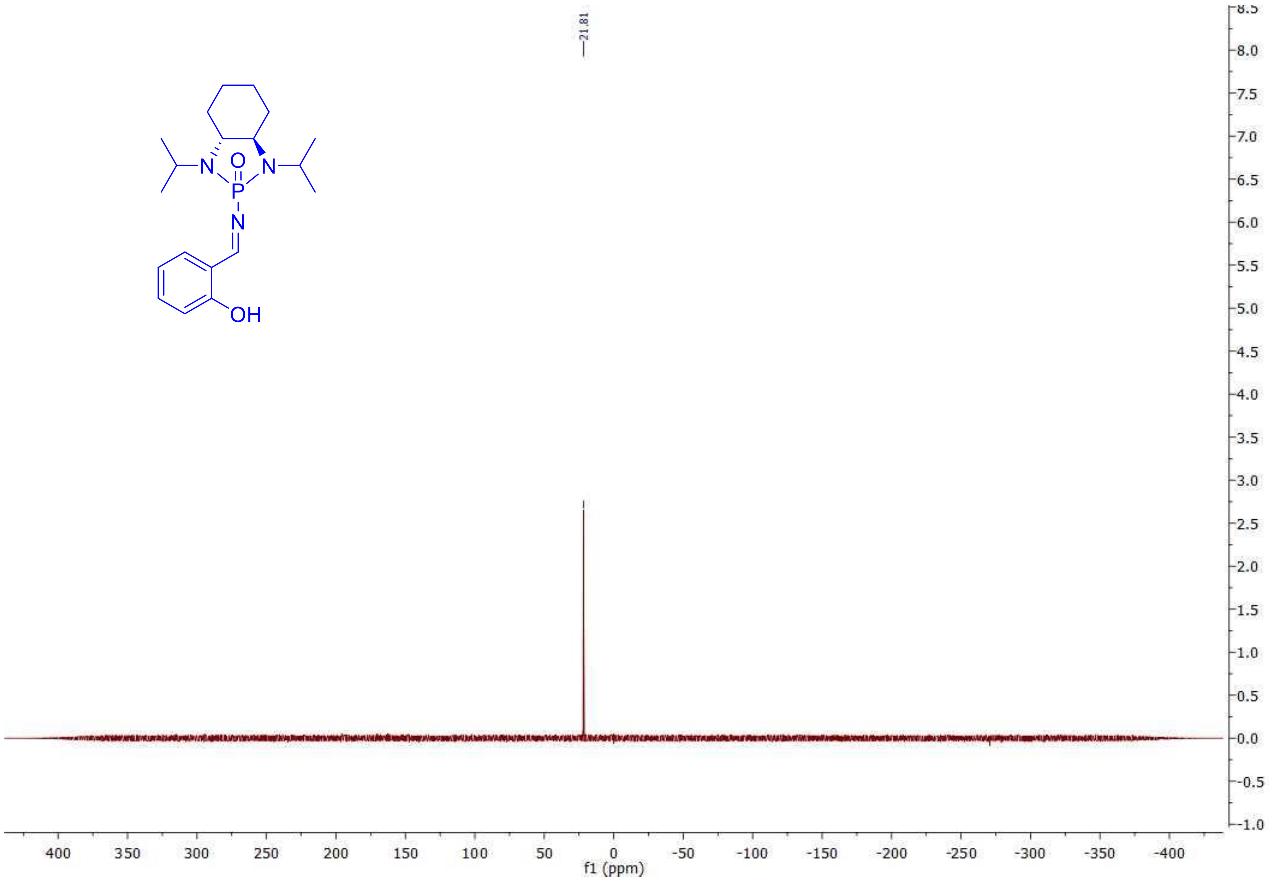

^{31}P NMR of imine **1a** ($CDCl_3$, 162 Hz)

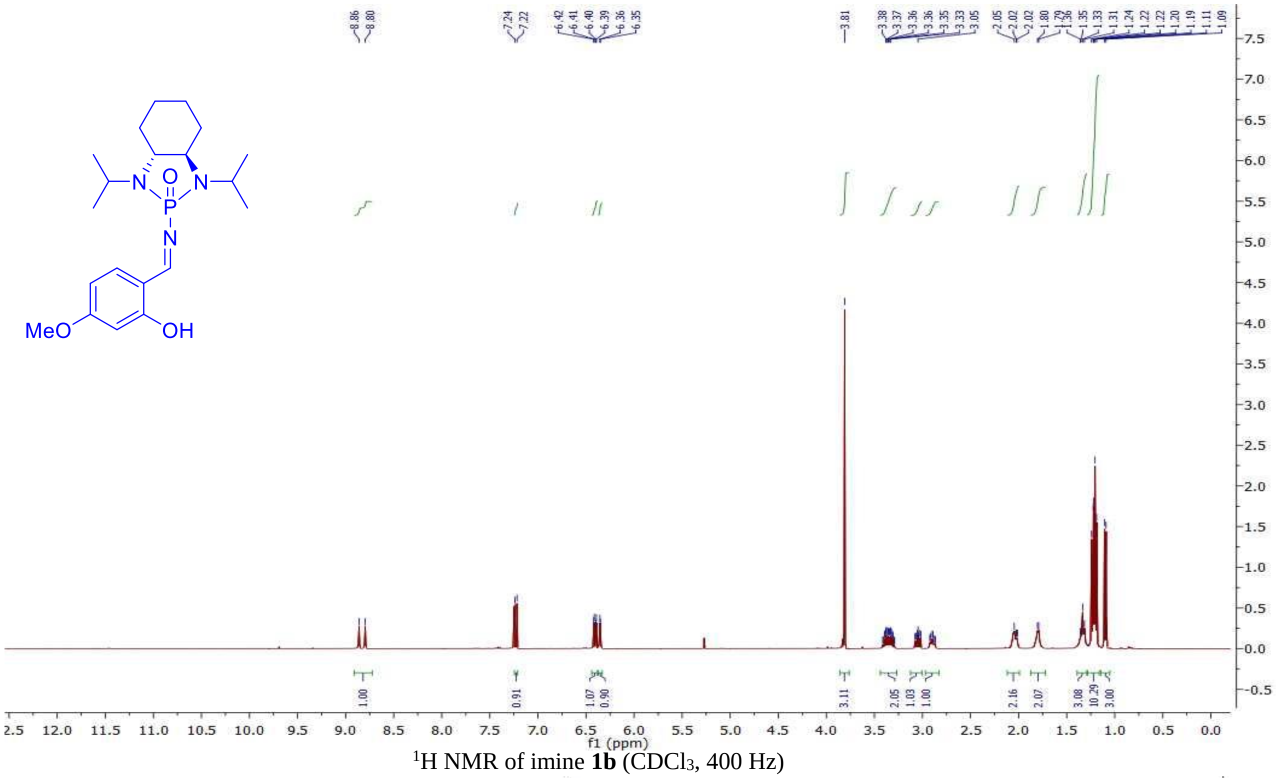

^{1}H NMR of imine **1b** ($CDCl_3$, 400 Hz)

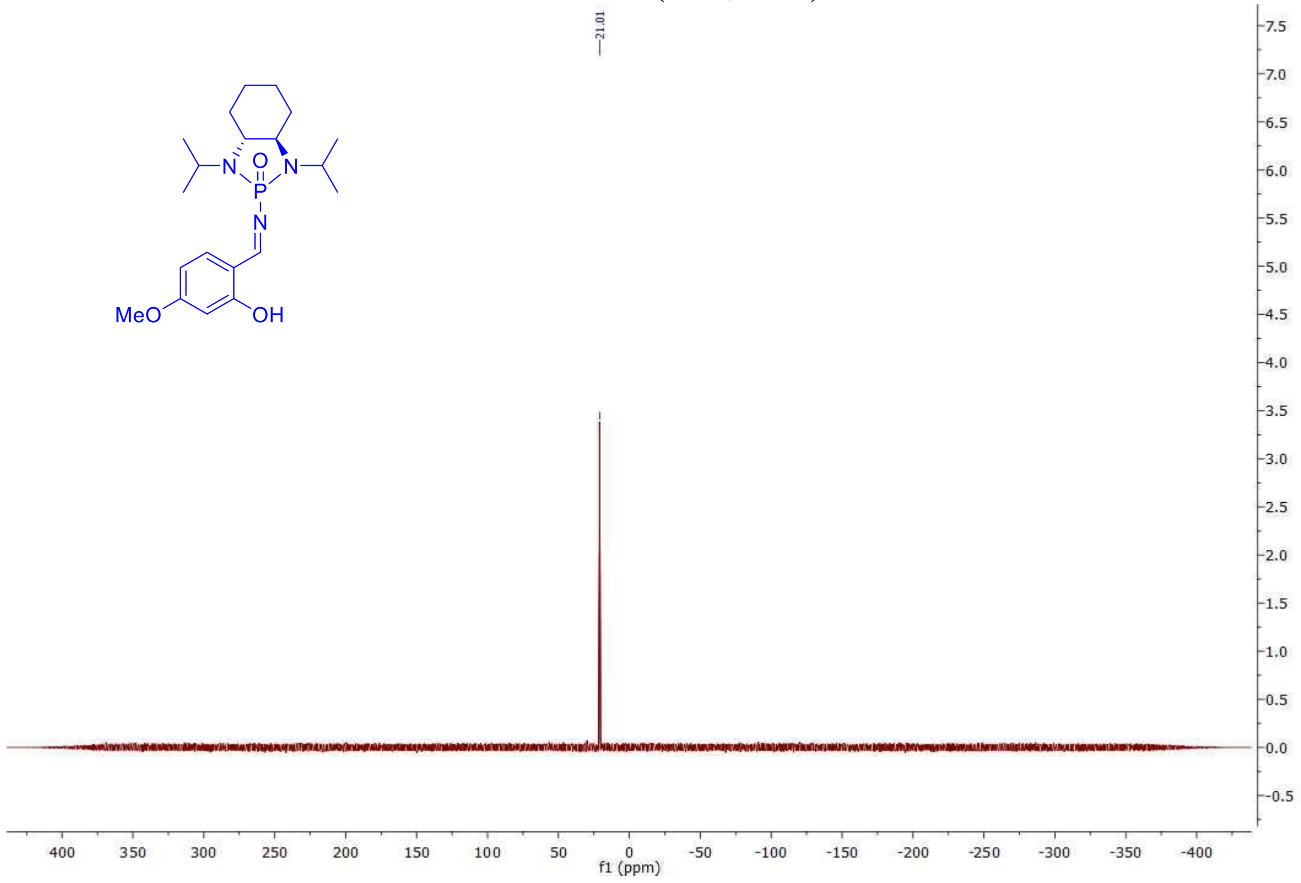

^{13}C NMR of imine **1b** ($CDCl_3$, 100 Hz)

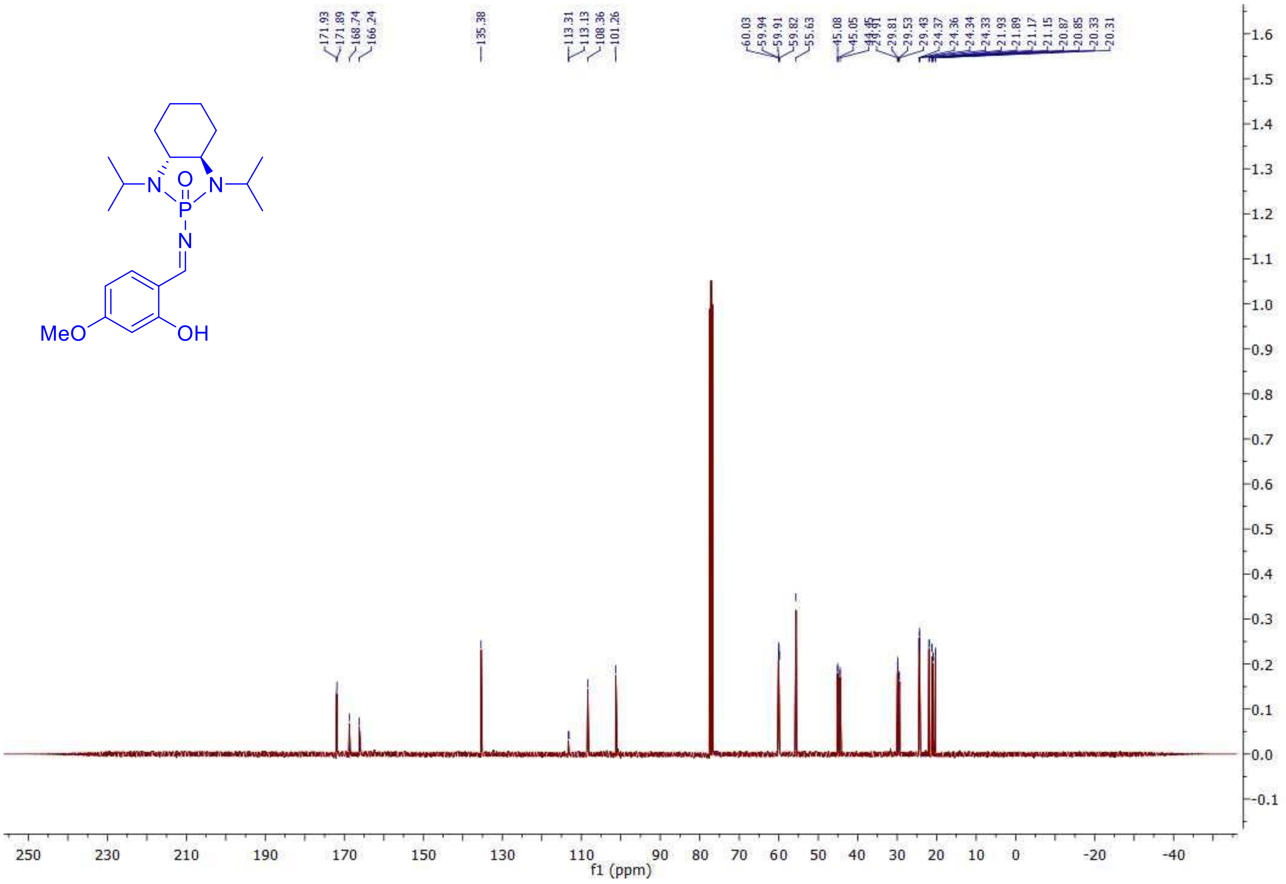

^{31}P NMR of imine **1b** ($CDCl_3$, 162 Hz)

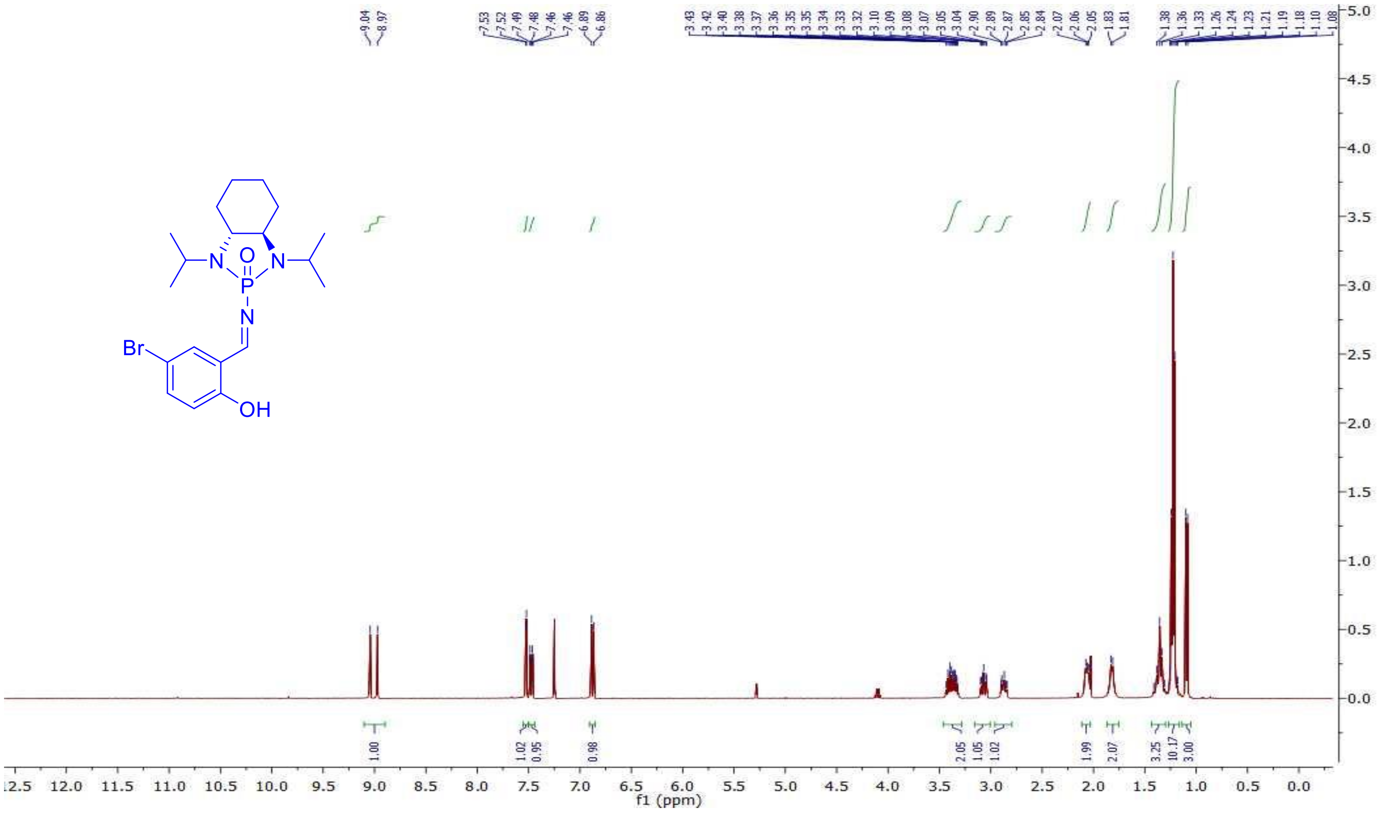

^{1}H NMR of imine **1c** ($CDCl_3$, 400 Hz)

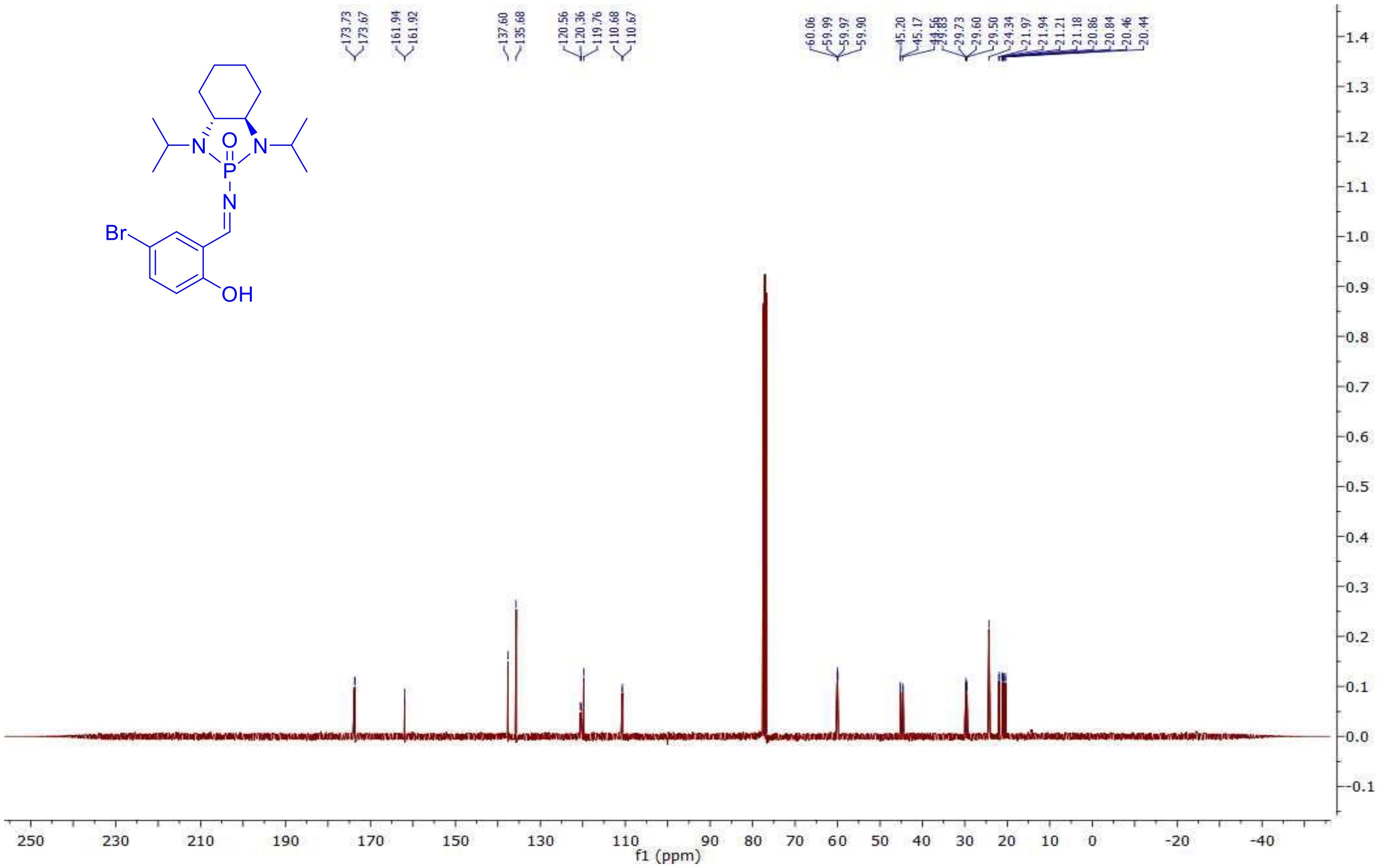

^{13}C NMR of imine **1c** ($CDCl_3$, 100 Hz)

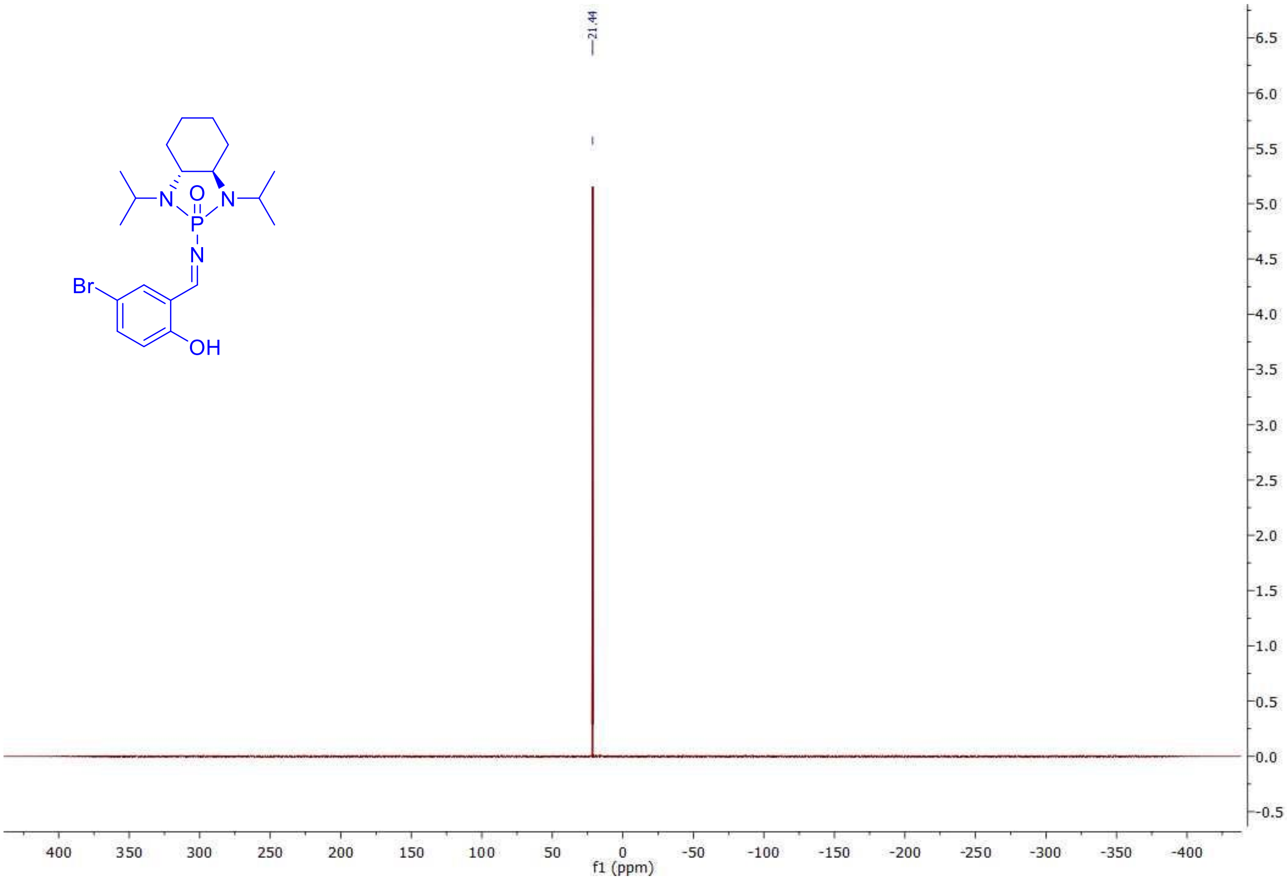

^{31}P NMR of imine **1c** ($CDCl_3$, 162 Hz)

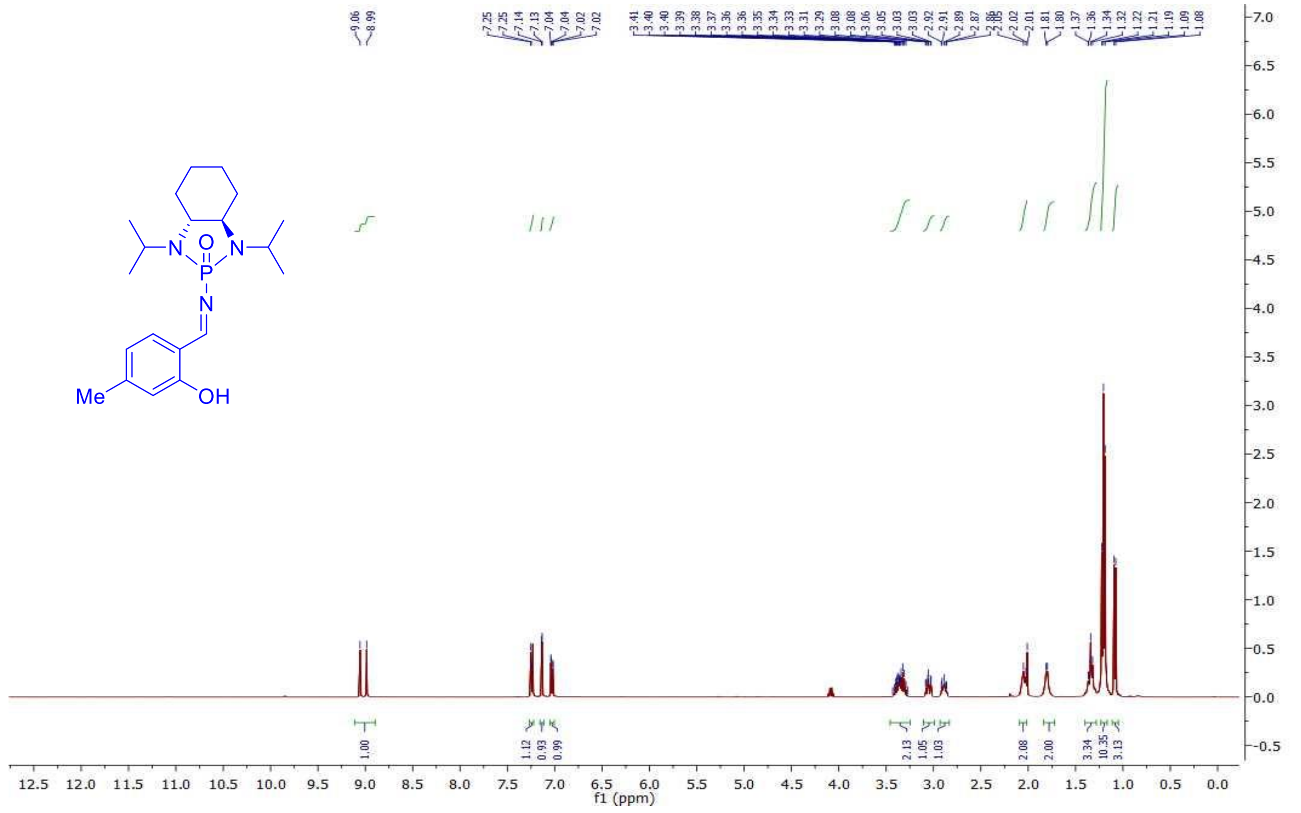

^{1}H NMR of imine **1d** ($CDCl_3$, 400 Hz)

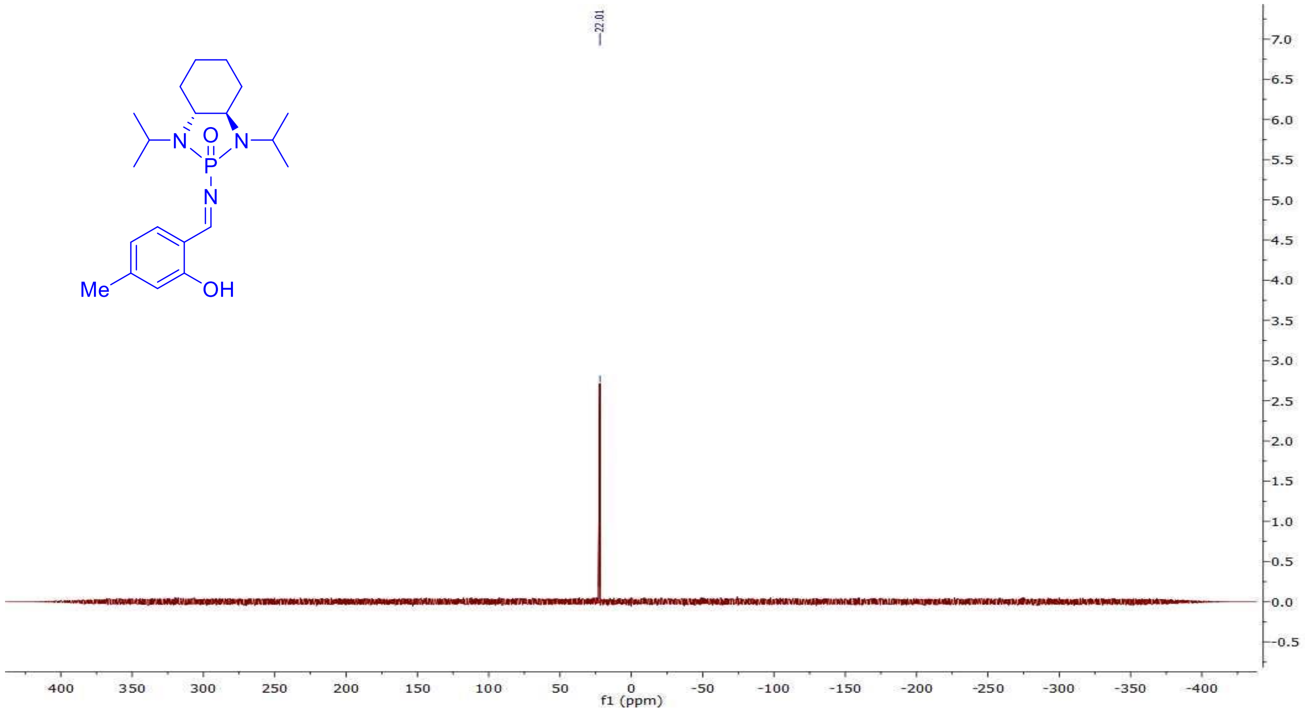

^{13}C NMR of imine **1d** ($CDCl_3$, 100 Hz)

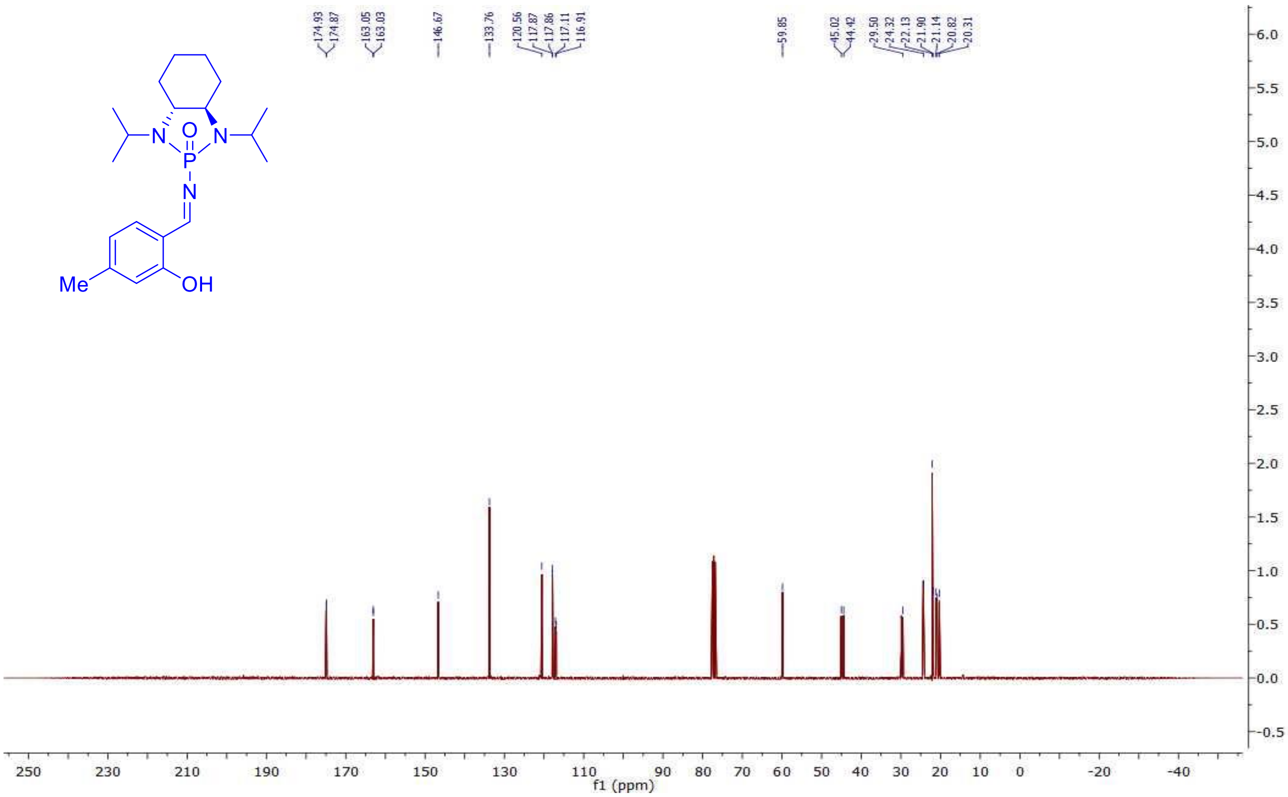

^{31}P NMR of imine **1d** ($CDCl_3$, 162 Hz)

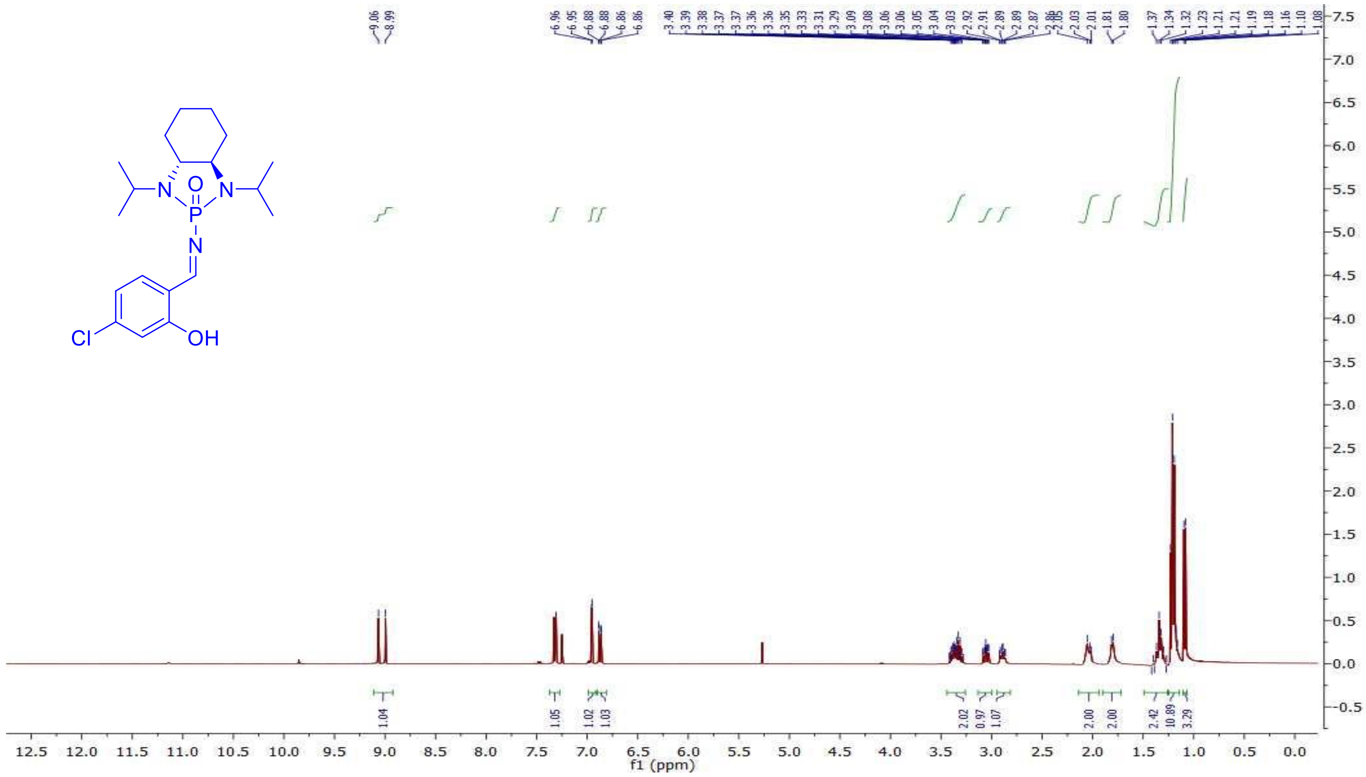

^{1}H NMR of imine **1e** ($CDCl_3$, 400 Hz)

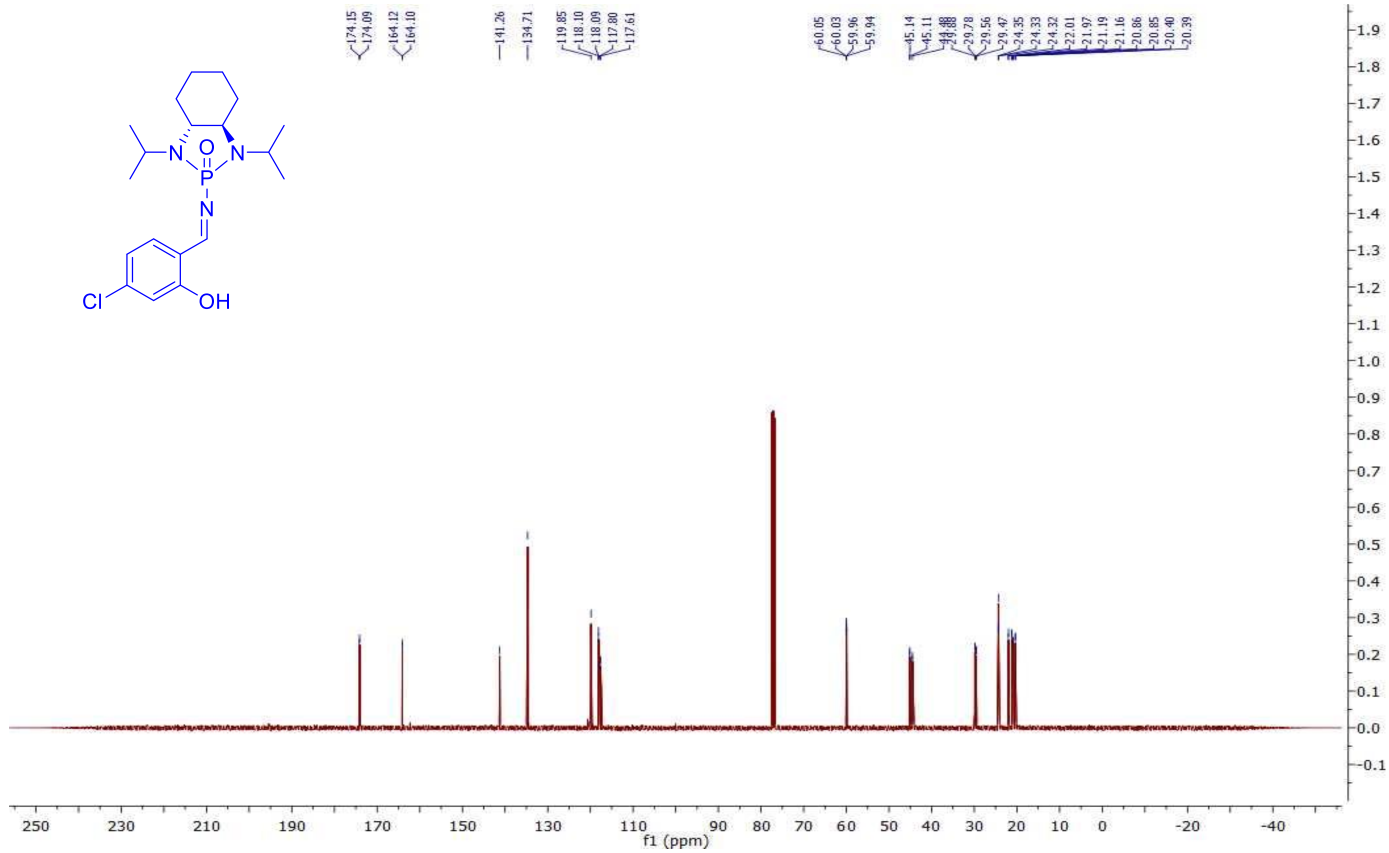

^{13}C NMR of imine **1e** ($CDCl_3$, 100 Hz)

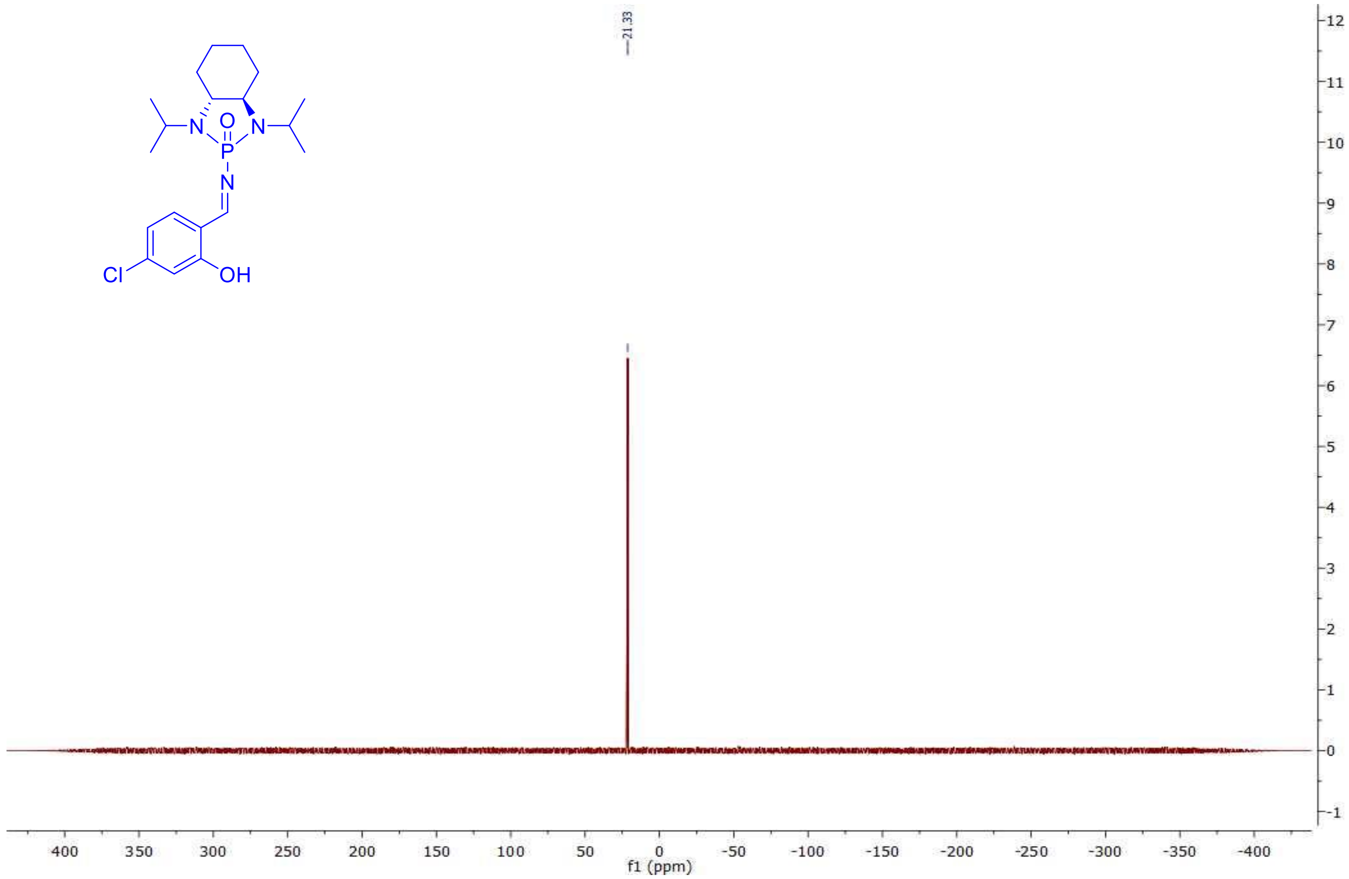

^{31}P NMR of imine **1e** ($CDCl_3$, 162 Hz)

Synthesis and characterization of 2,3-dihydrobenzofuran (3a-3e): Into an oven-dried round bottom flask, flushed and protected by argon, salicyl N-phosphonyl imine (0.15 mmol) was dissolved in THF. After 5 minutes, potassium phosphate (0.3 mmol) was added in one portion and the reaction stir at room temperature for an hour. Diethyl bromo malonate (0.3 mmol) dissolved in Ethanol was added dropwise and the reaction kept at room temperature for 24 hours. Then the reaction was quenched with 3 ml water and extract with ethyl acetate and dried over sodium sulfate. The product was separated by addition of hexanes to precipitate the product as white solid.

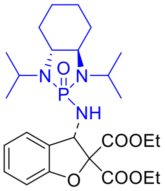

THF: Ethanol	S:R ratio
6.5:1	66:34
4.5:1	65:35
2.5:1	59:41
1:1	54:46
1:2.5	35:65
1:4.5	31:69
1:6.5	29:71

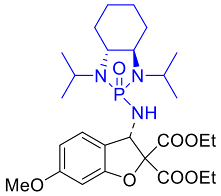

THF: Ethanol	S:R ratio
6.5:1	58:42
4.5:1	54:46
2.5:1	51:49
1:1	40:60
1:2.5	32:68
1:4.5	31:69
1:6.5	30:70

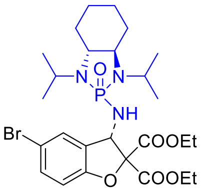

THF: Ethanol	S:R ratio
6.5:1	58:42
4.5:1	57:43
2.5:1	54:46
1:1	44:56
1:2.5	35:65
1:4.5	32:68
1:6.5	30:70

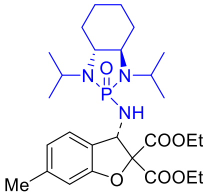

THF: Ethanol	S:R ratio
6.5:1	65:35
4.5:1	65:35
2.5:1	57:43
1:1	45:55
1:2.5	35:65
1:4.5	31:69
1:6.5	30:70

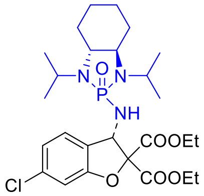

THF: Ethanol	S:R ratio
6.5:1	54:46
4.5:1	53:47
2.5:1	51:49
1:1	44:56
1:2.5	32:68
1:4.5	29:71
1:6.5	29:71

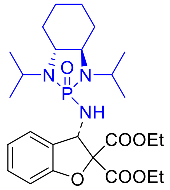

THF:EtOH (6.5:1) = Ratio S:R (66:34)

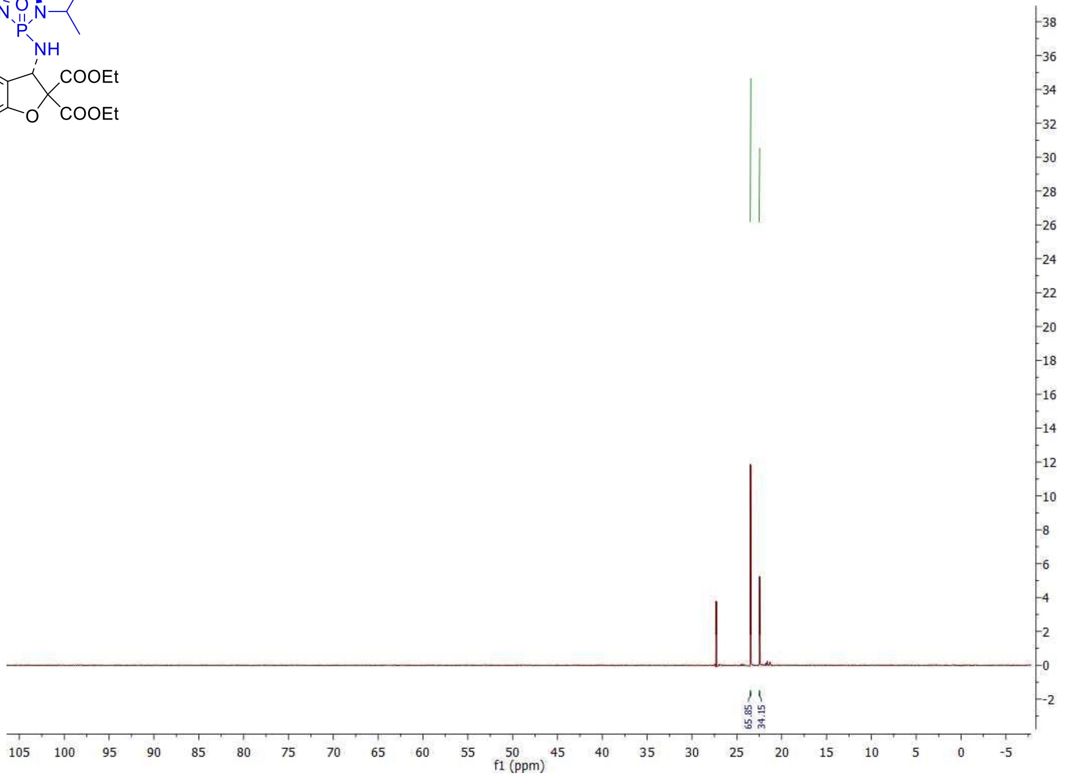

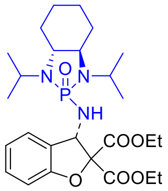

THF:EtOH (4.5:1) = Ratio S:R (65:35)

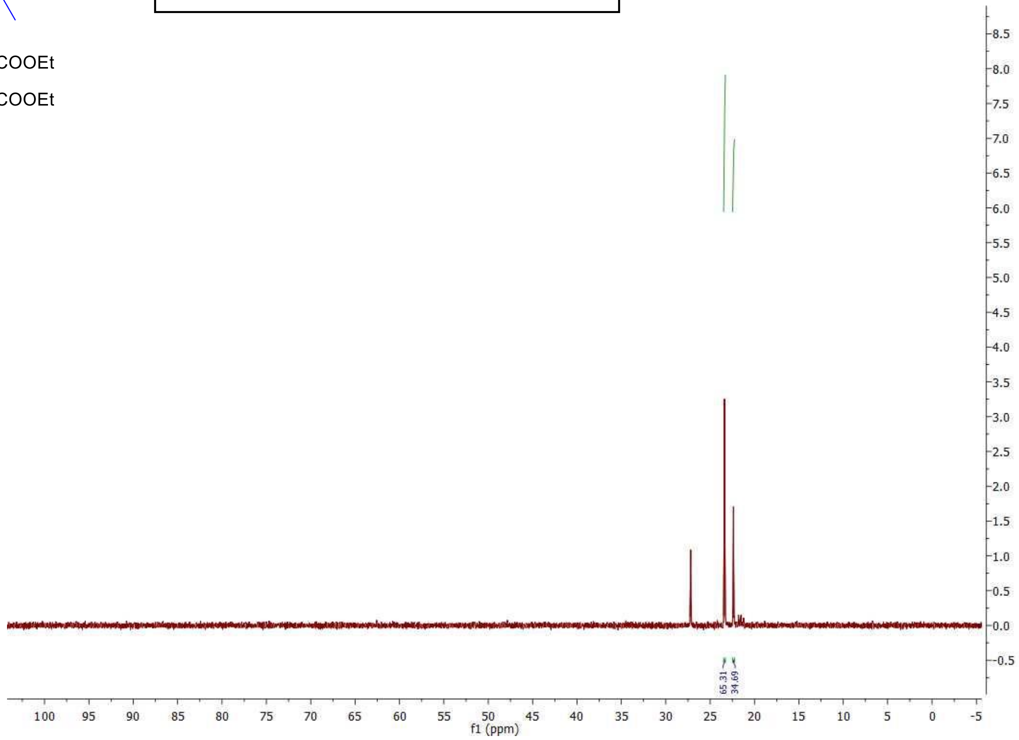

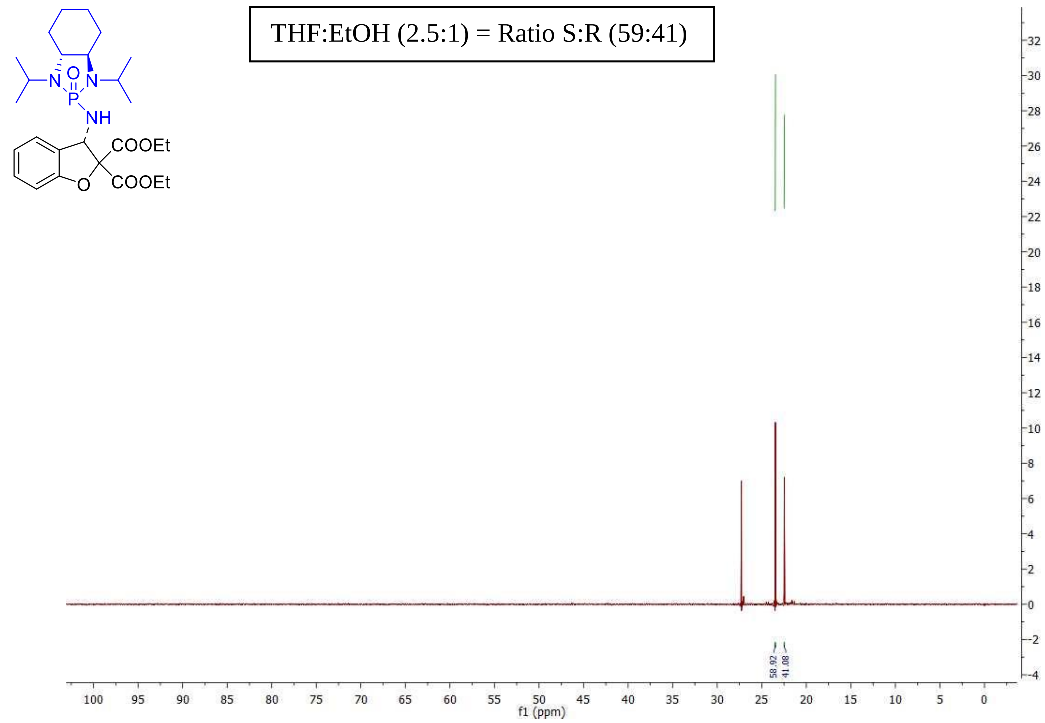
N
O
P
N
NH
COOEt
O
COOEt
THF:EtOH (2.5:1) = Ratio S:R (59:41)
58.92
41.08
100 95 90 85 80 75 70 65 60 55 50 45 40 35 30 25 20 15 10 5 0
f1 (ppm)

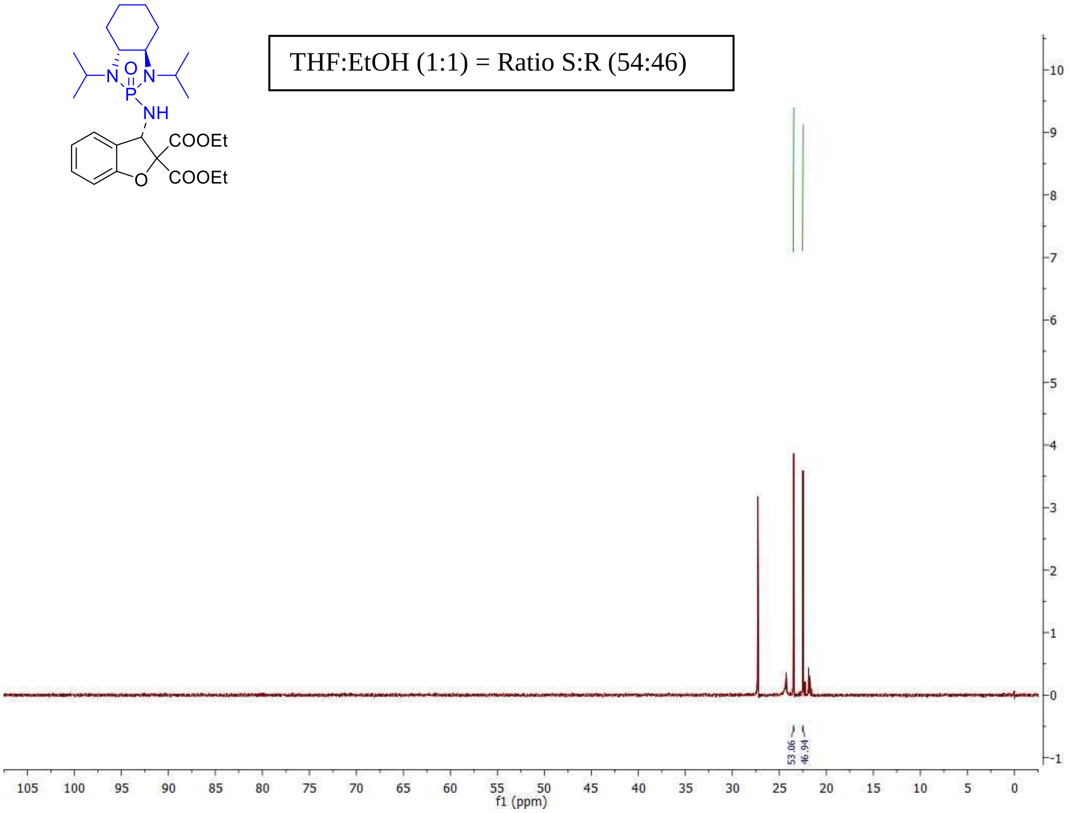
N
O
P
N
NH
COOEt
O
COOEt
THF:EtOH (1:1) = Ratio S:R (54:46)
53.06
46.94
105 100 95 90 85 80 75 70 65 60 55 50 45 40 35 30 25 20 15 10 5 0
f1 (ppm)

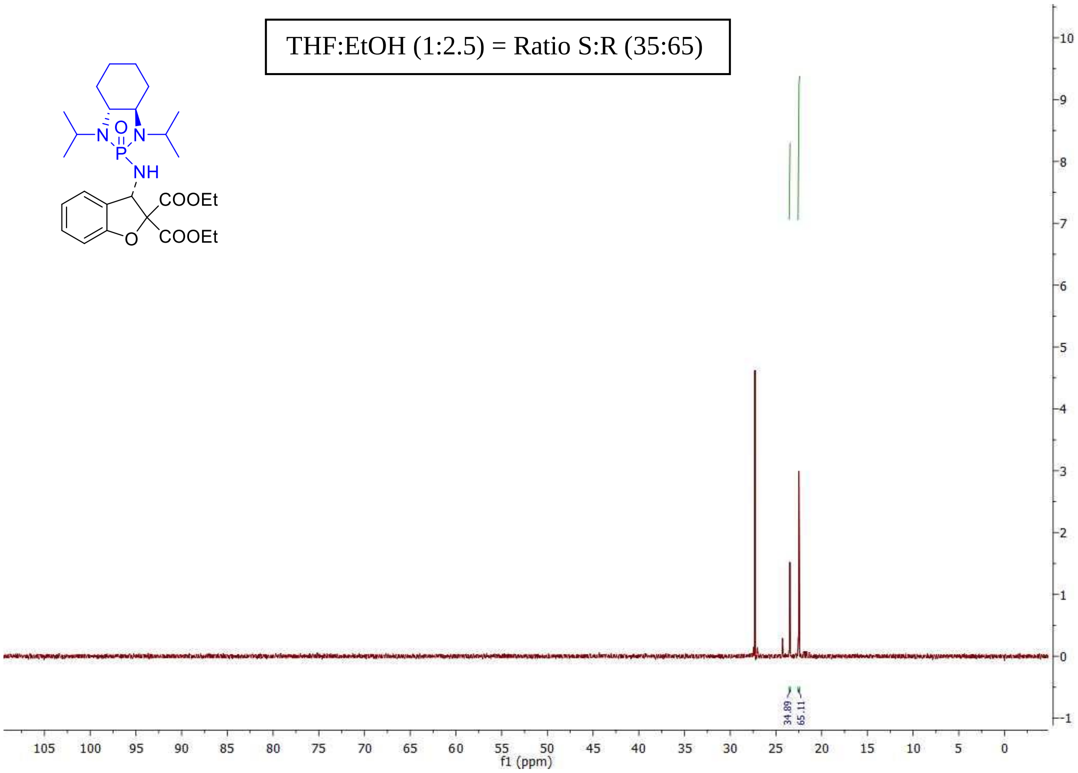

THF:EtOH (1:2.5) = Ratio S:R (35:65)
N
O
P
N
NH
COOEt
COOEt
O
34.89
65.11
f1 (ppm)

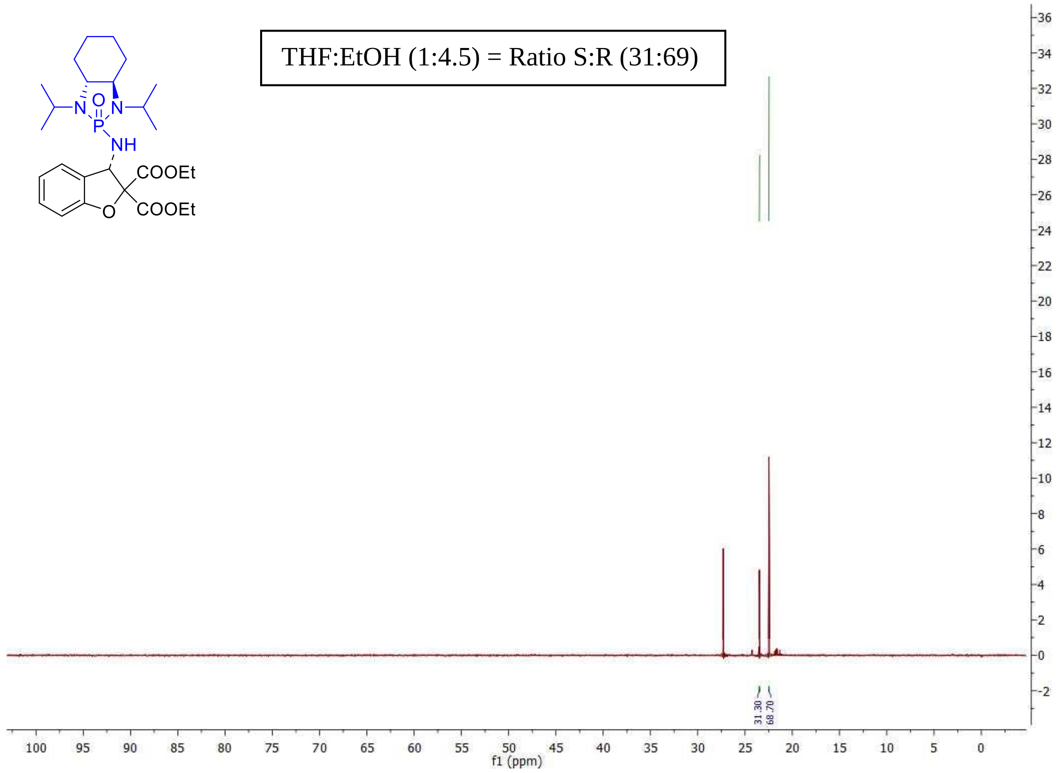

THF:EtOH (1:4.5) = Ratio S:R (31:69)
N
O
P
N
NH
COOEt
COOEt
O
31.30
68.70
f1 (ppm)

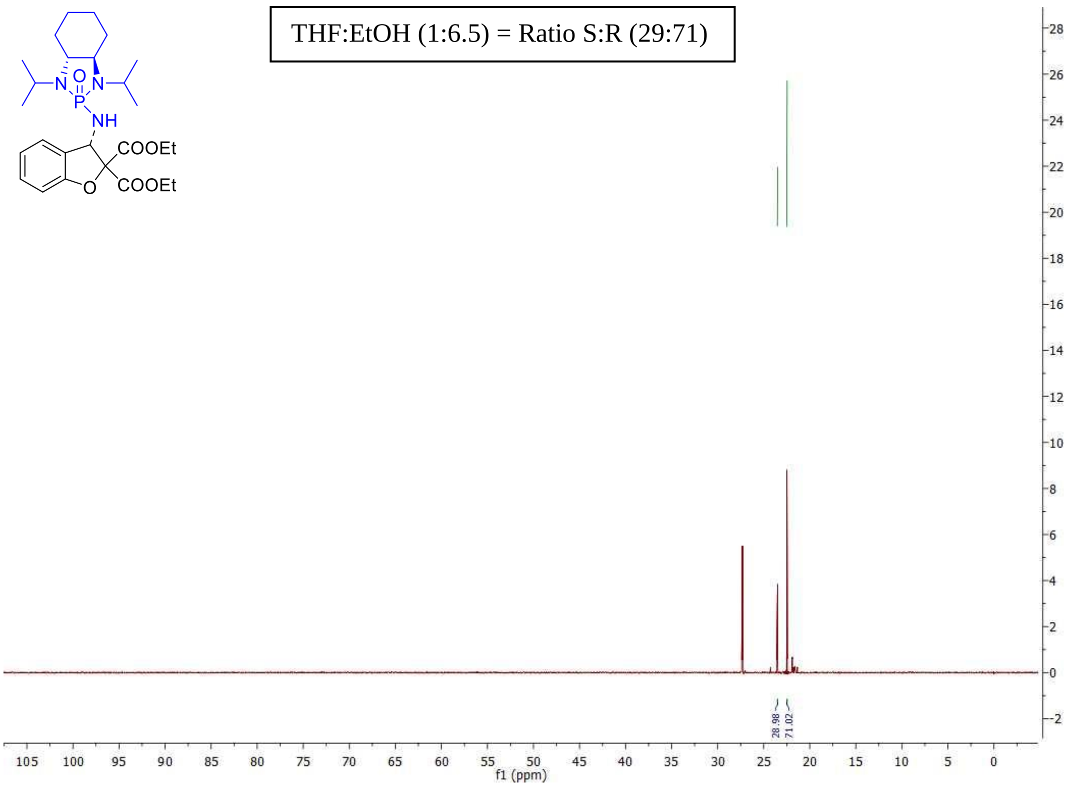
THF:EtOH (1:6.5) = Ratio S:R (29:71)
NH
COOEt
COOEt
O
N
P
N
28.98
71.02
f1 (ppm)
105 100 95 90 85 80 75 70 65 60 55 50 45 40 35 30 25 20 15 10 5 0
28 26 24 22 20 18 16 14 12 10 8 6 4 2 0 -2

THF:EtOH (6.5:1) = Ratio S:R (58:42)

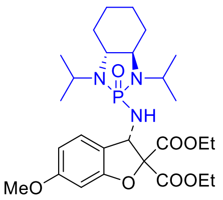

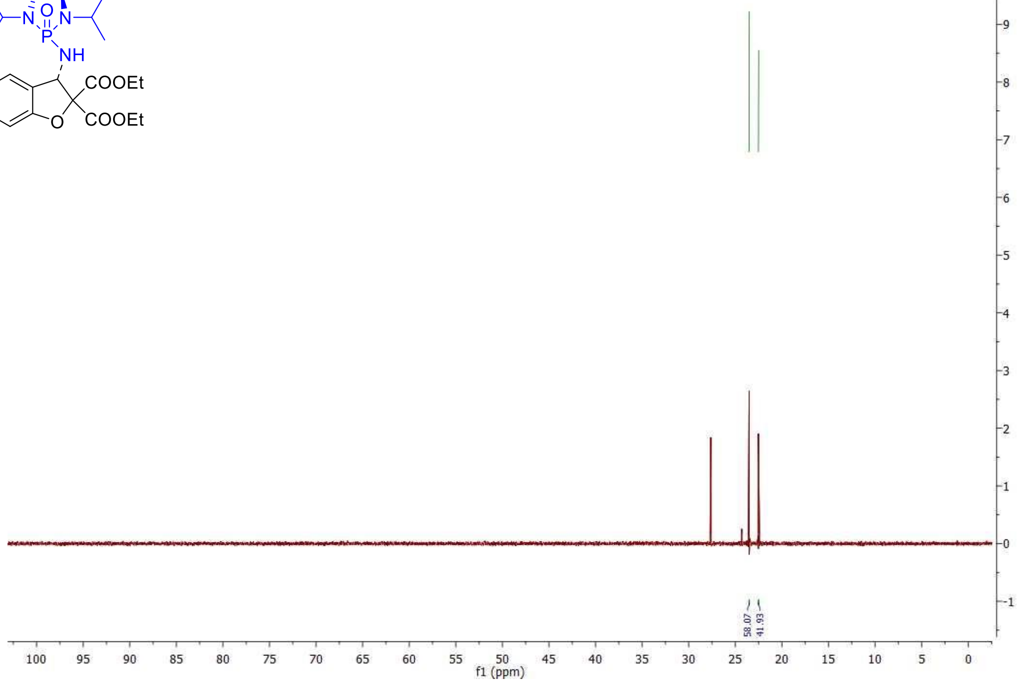

THF:EtOH (4.5:1) = Ratio S:R (54:46)

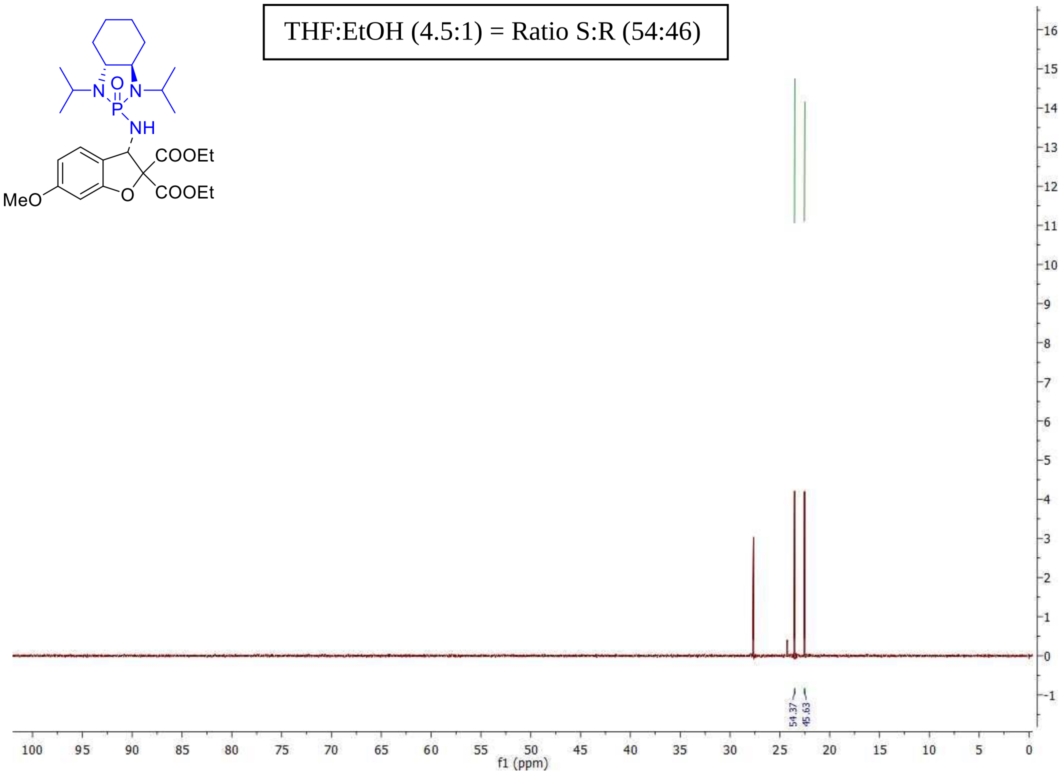

THF:EtOH (2.5:1) = Ratio S:R (51:49)

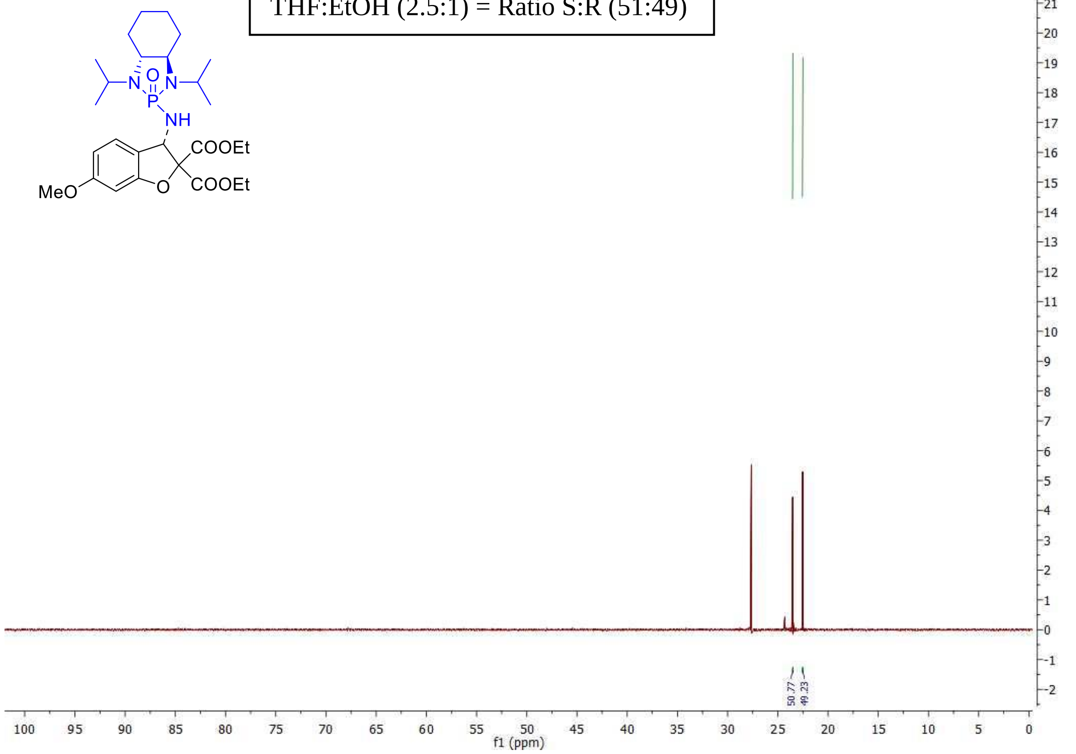

THF:EtOH (1:1) = Ratio S:R (40:60)

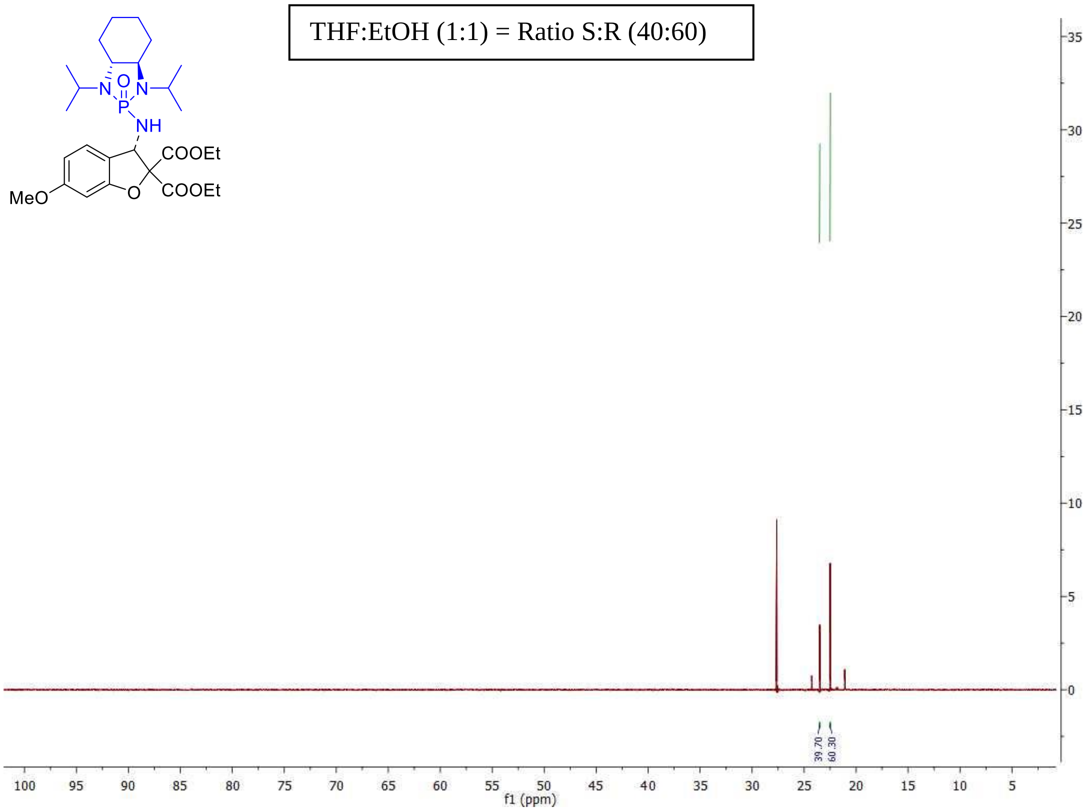

THF:EtOH (1:2.5) = Ratio S:R (32:68)

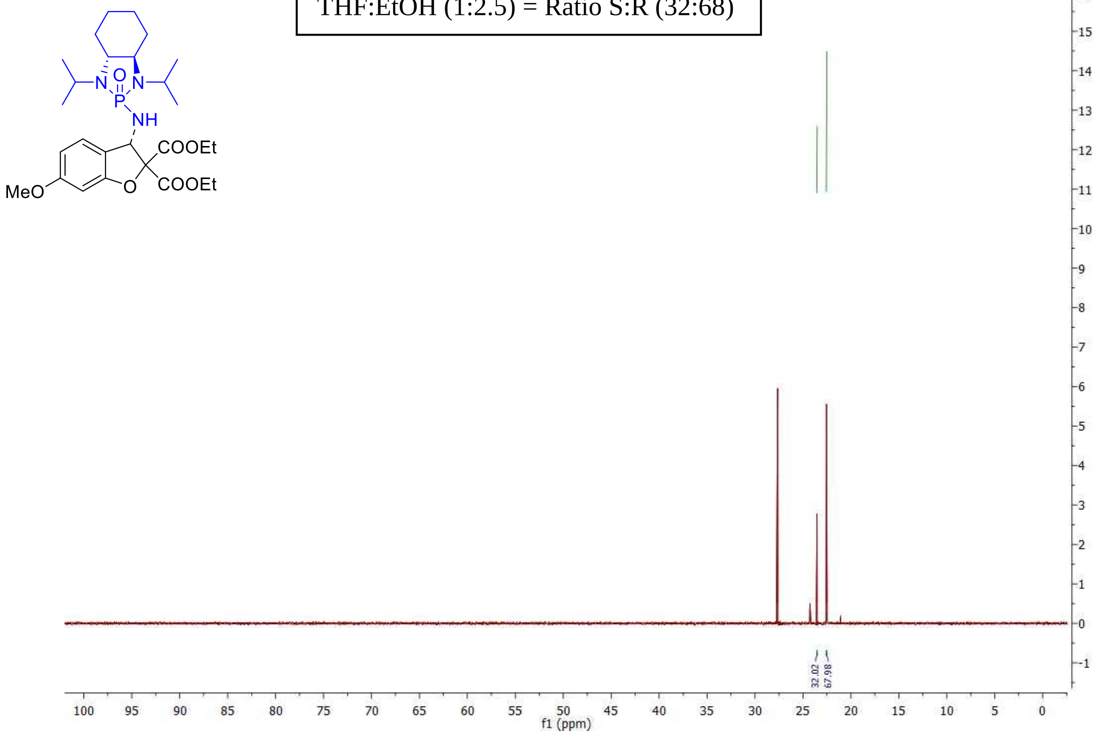

THF:EtOH (1:4.5) = Ratio S:R (31:69)

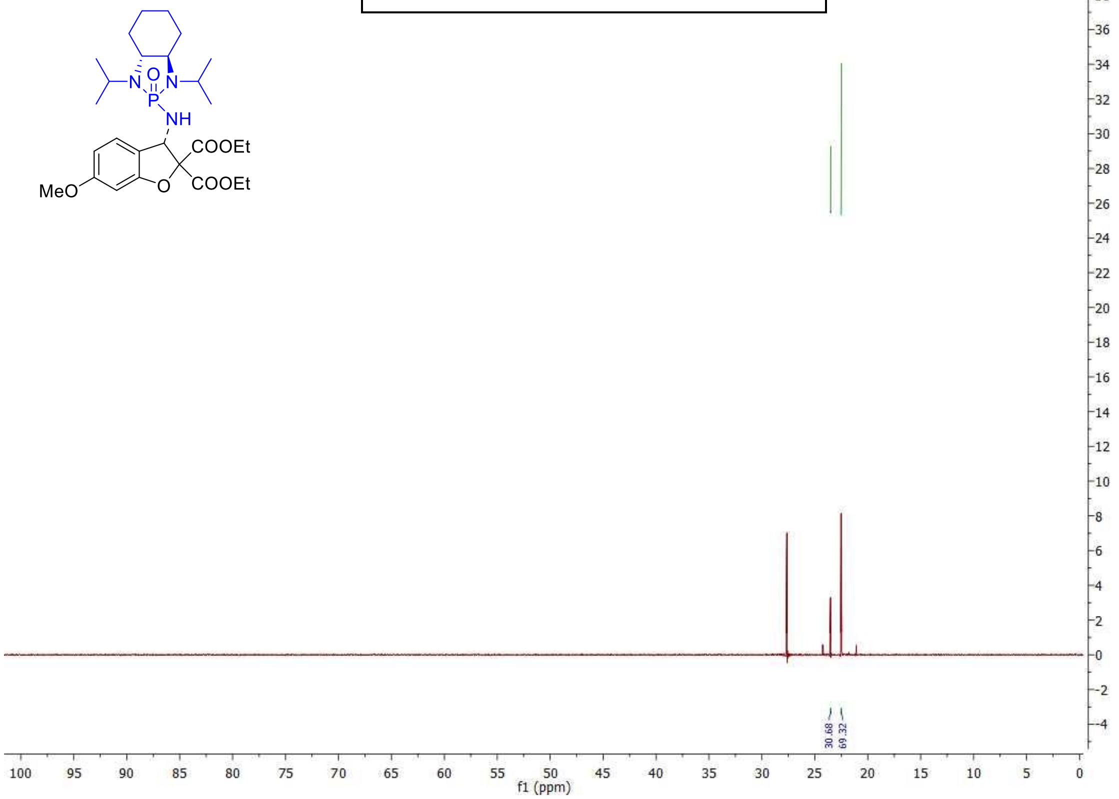

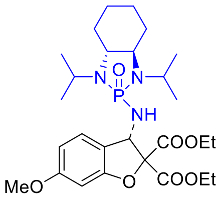

THF:EtOH (1:6.5) = Ratio S:R (30:70)

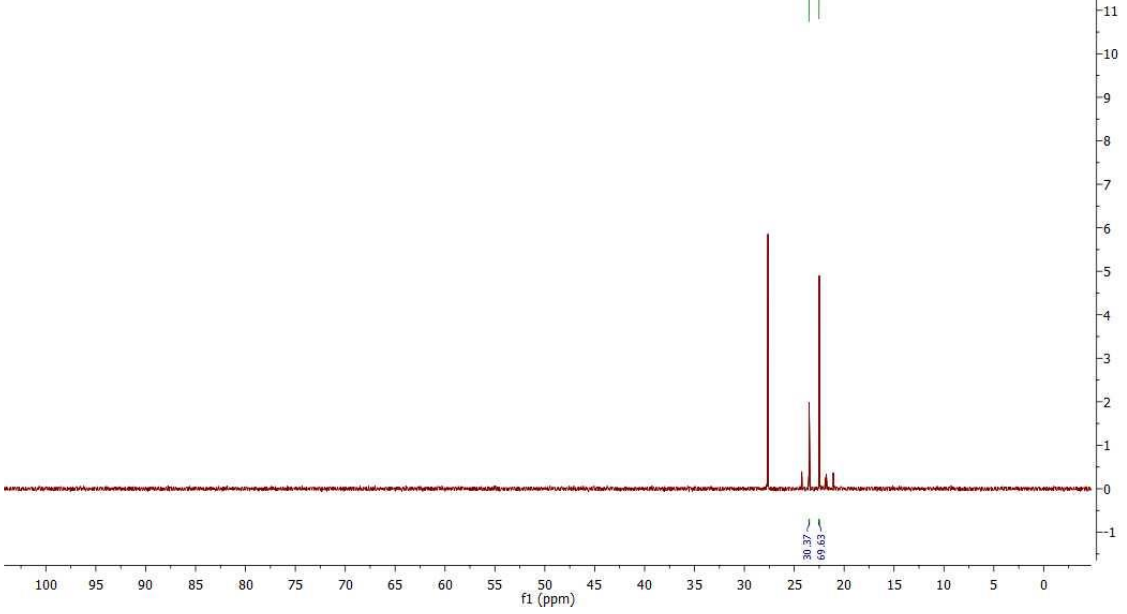

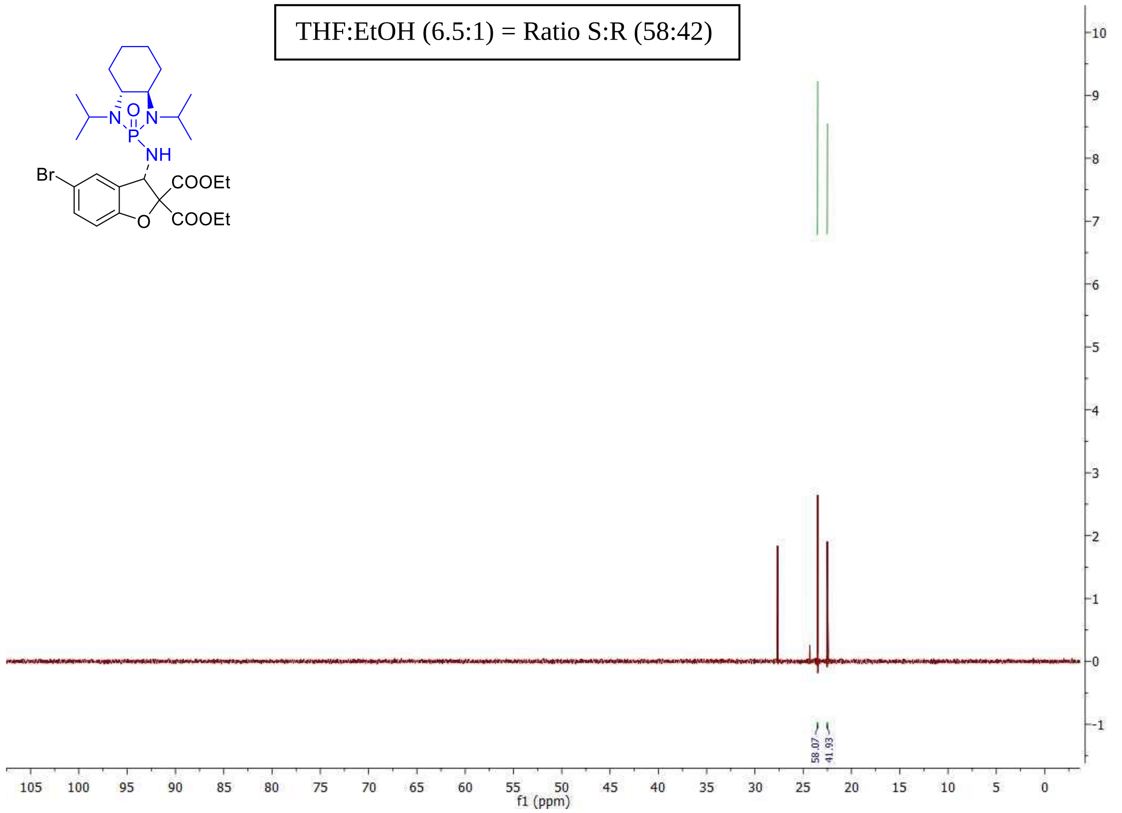
THF:EtOH (6.5:1) = Ratio S:R (58:42)
Br
NH
COOEt
COOEt
58.07
41.93
f1 (ppm)

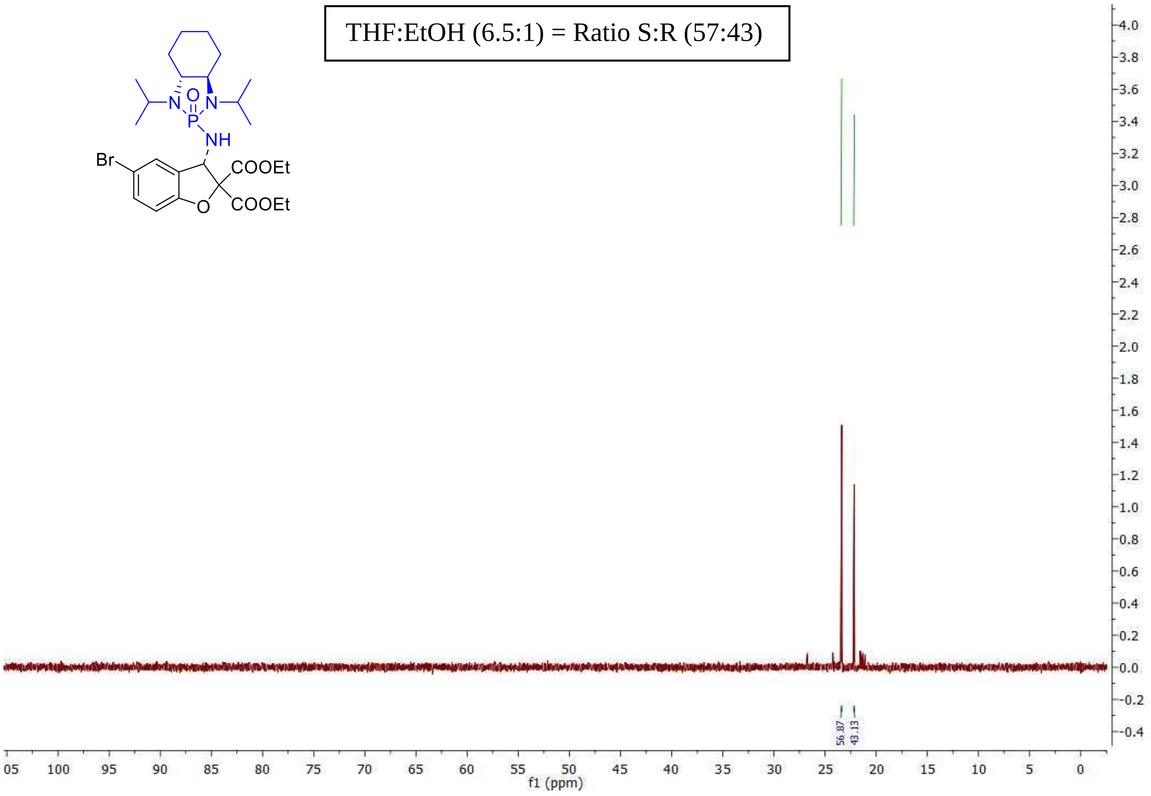
THF:EtOH (6.5:1) = Ratio S:R (57:43)
Br
NH
COOEt
COOEt
56.87
43.13
f1 (ppm)

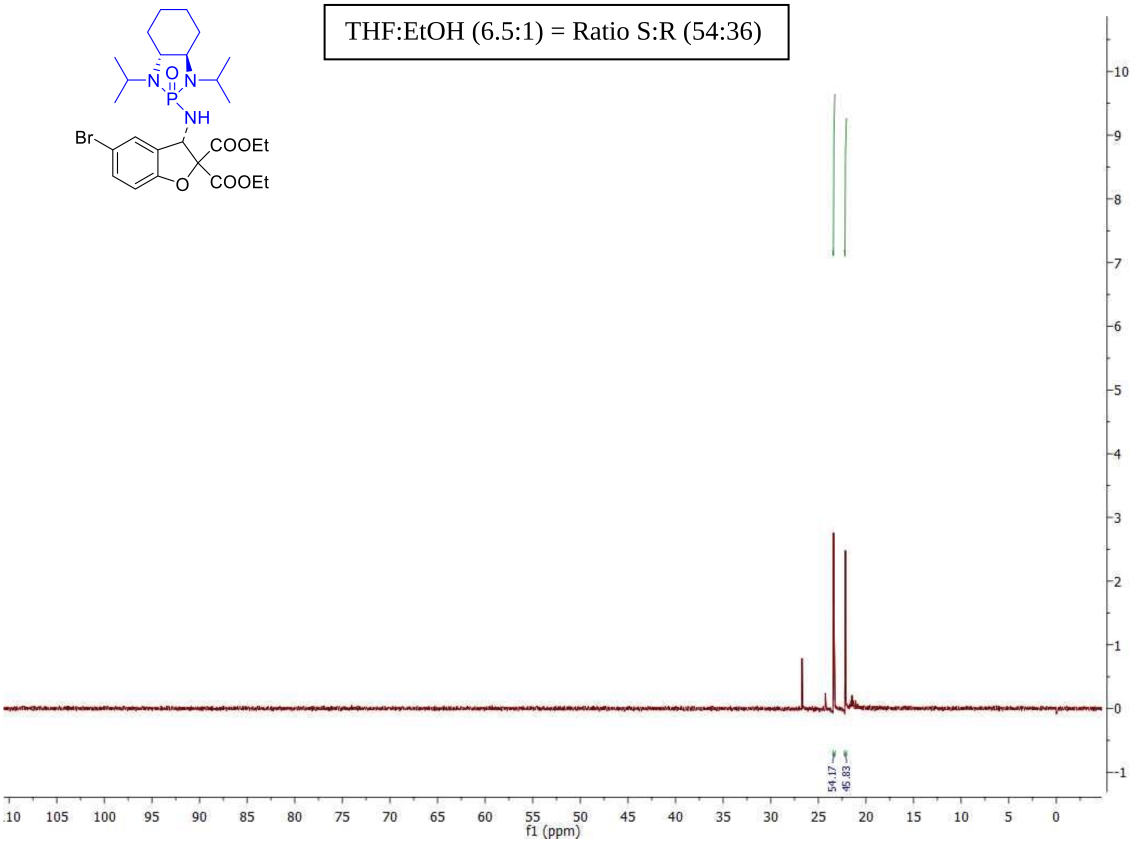
THF:EtOH (6.5:1) = Ratio S:R (54:36)
Br
NH
O
N
P
COOEt
COOEt
54.17
45.83
f1 (ppm)

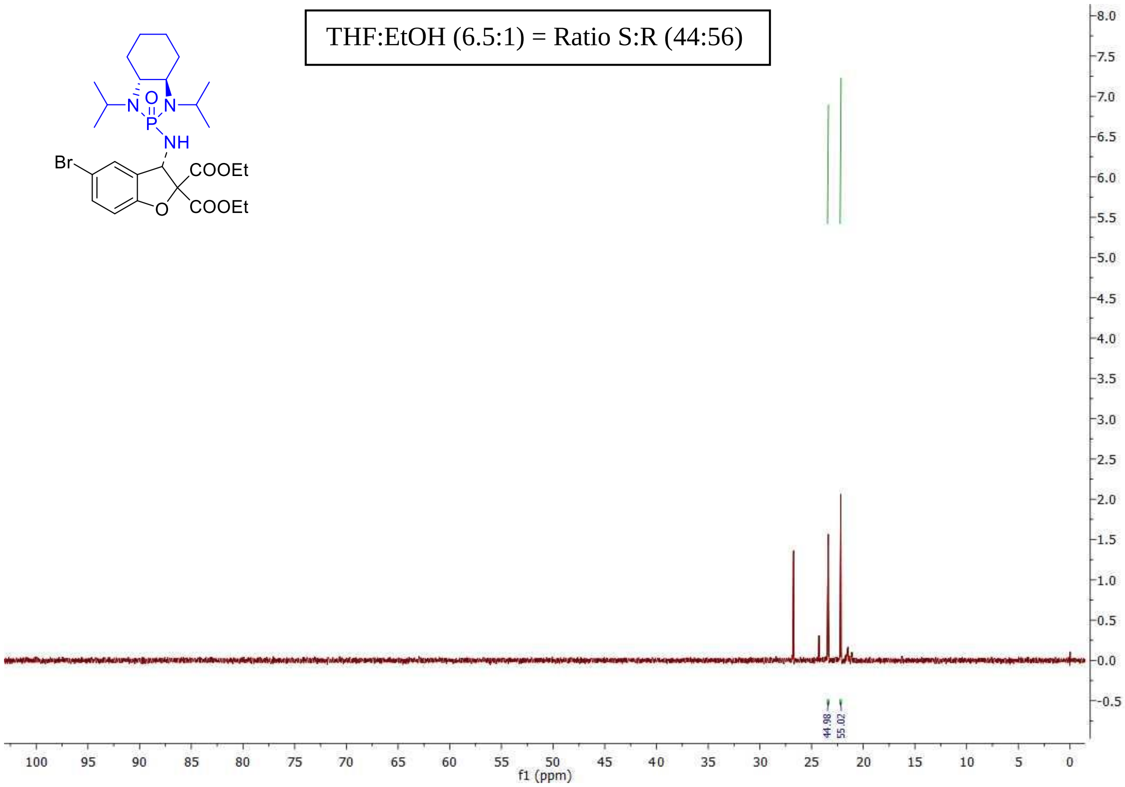
THF:EtOH (6.5:1) = Ratio S:R (44:56)
Br
NH
O
N
P
COOEt
COOEt
44.98
55.02
f1 (ppm)

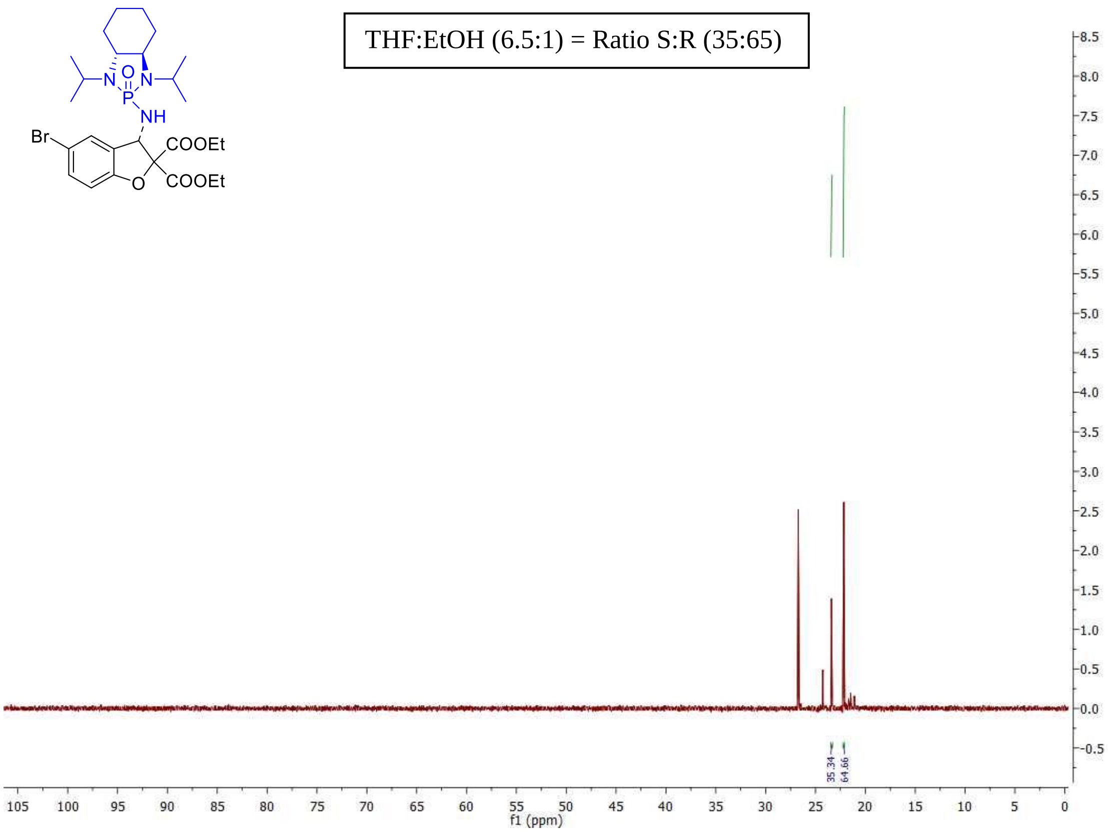
THF:EtOH (6.5:1) = Ratio S:R (35:65)
Br
COOEt
COOEt
O
N
P
NH
35.34
64.66
f1 (ppm)

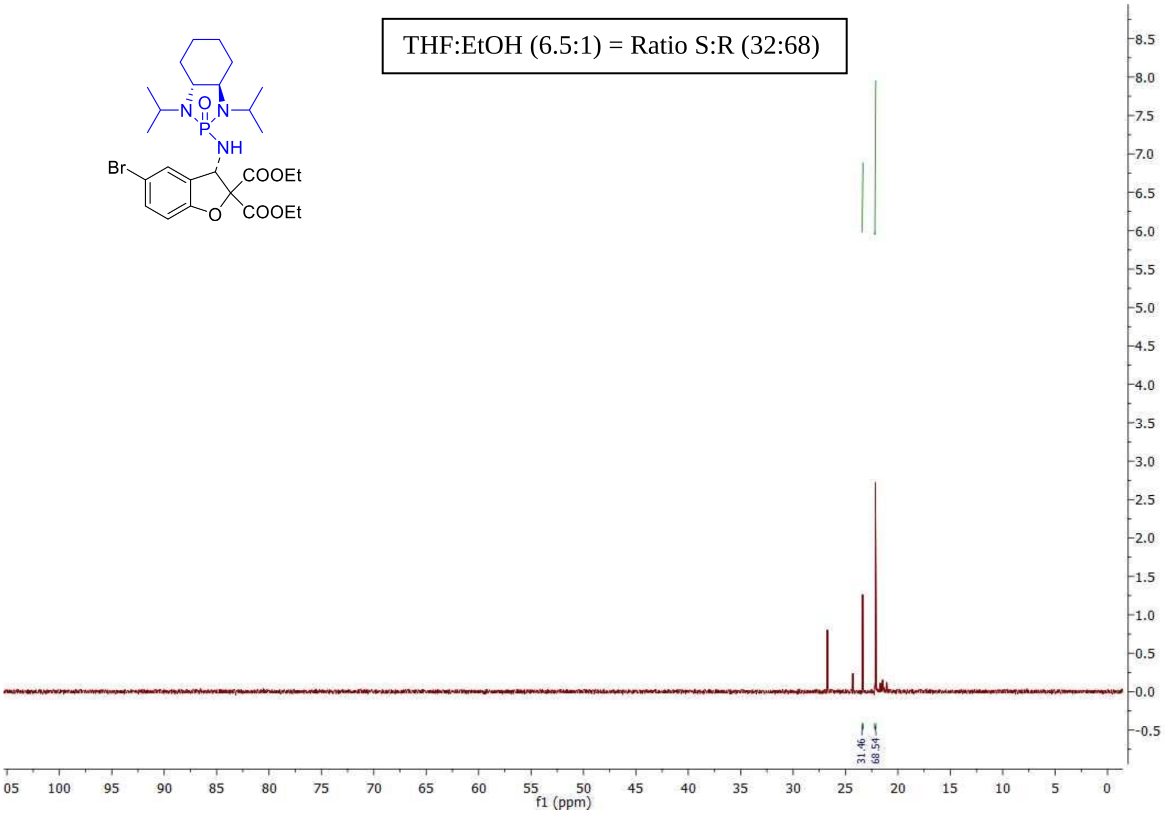
THF:EtOH (6.5:1) = Ratio S:R (32:68)
Br
COOEt
COOEt
O
N
P
NH
31.46
68.54
f1 (ppm)

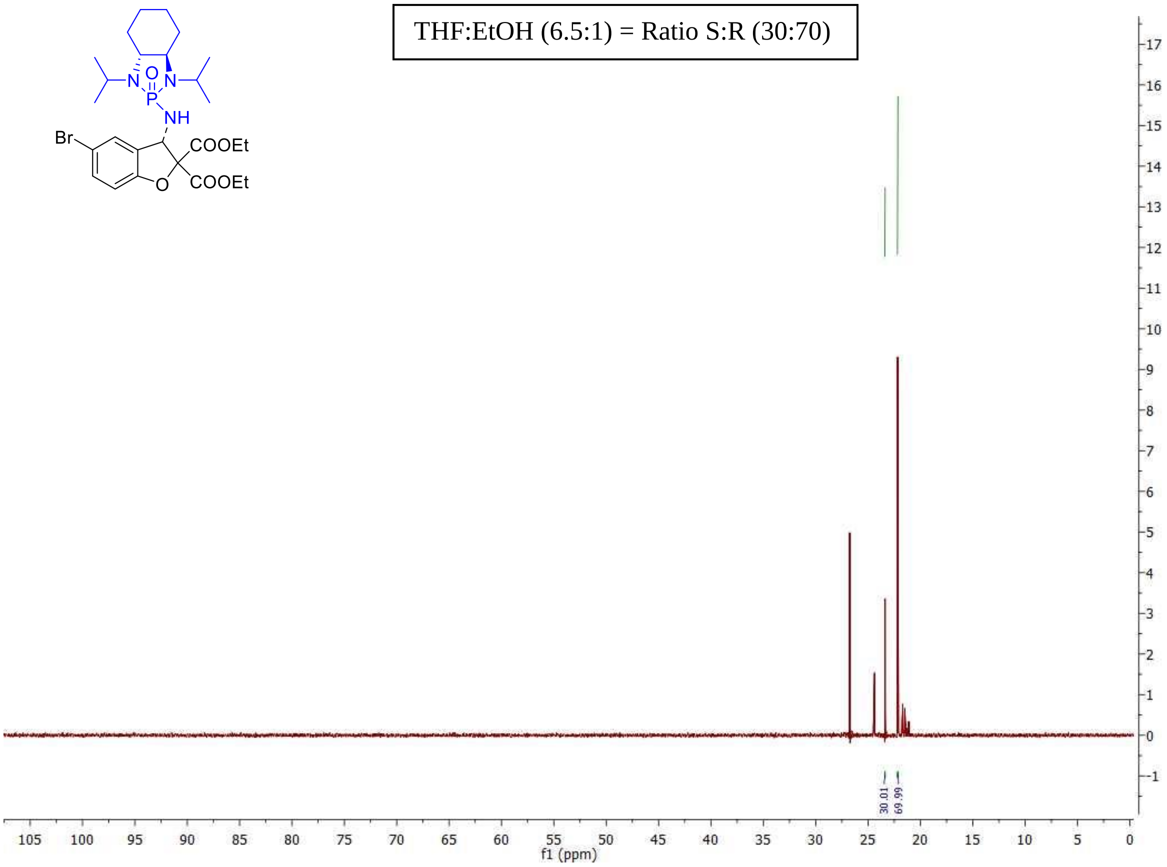
Br
NH
O
P
N
N
COOEt
COOEt
O
THF:EtOH (6.5:1) = Ratio S:R (30:70)
30.01
69.99
f1 (ppm)

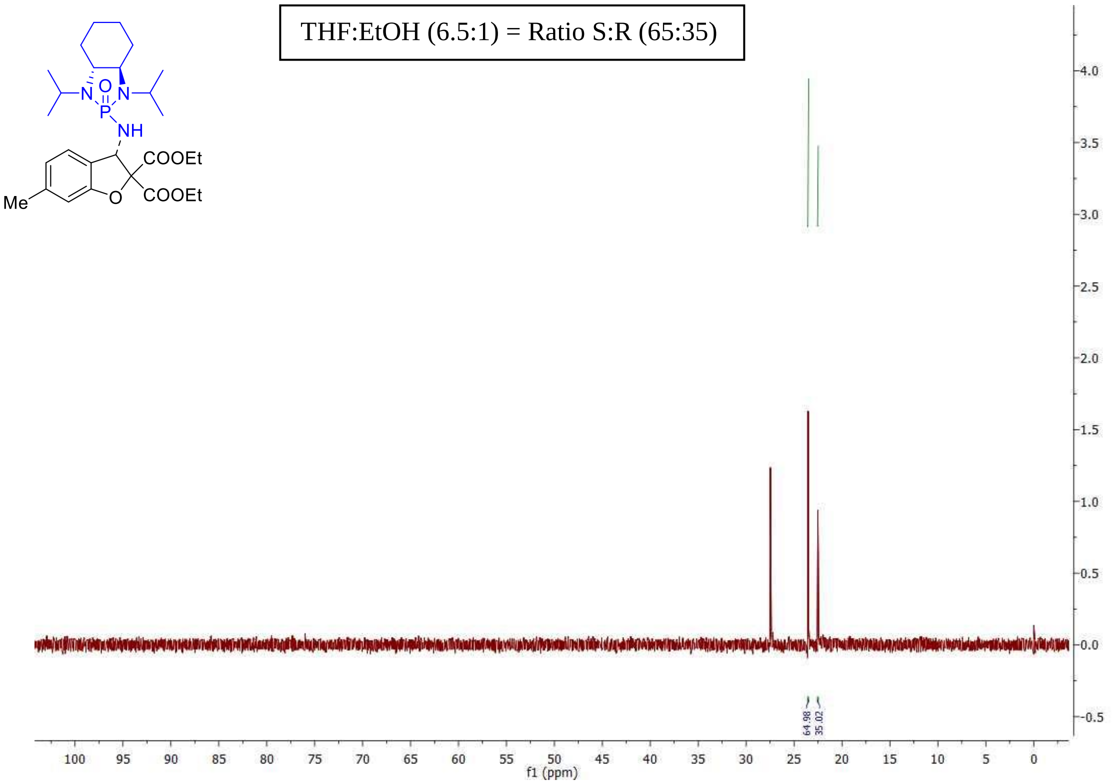
THF:EtOH (6.5:1) = Ratio S:R (65:35)
NH
COOEt
COOEt
Me
64.98
35.02
f1 (ppm)

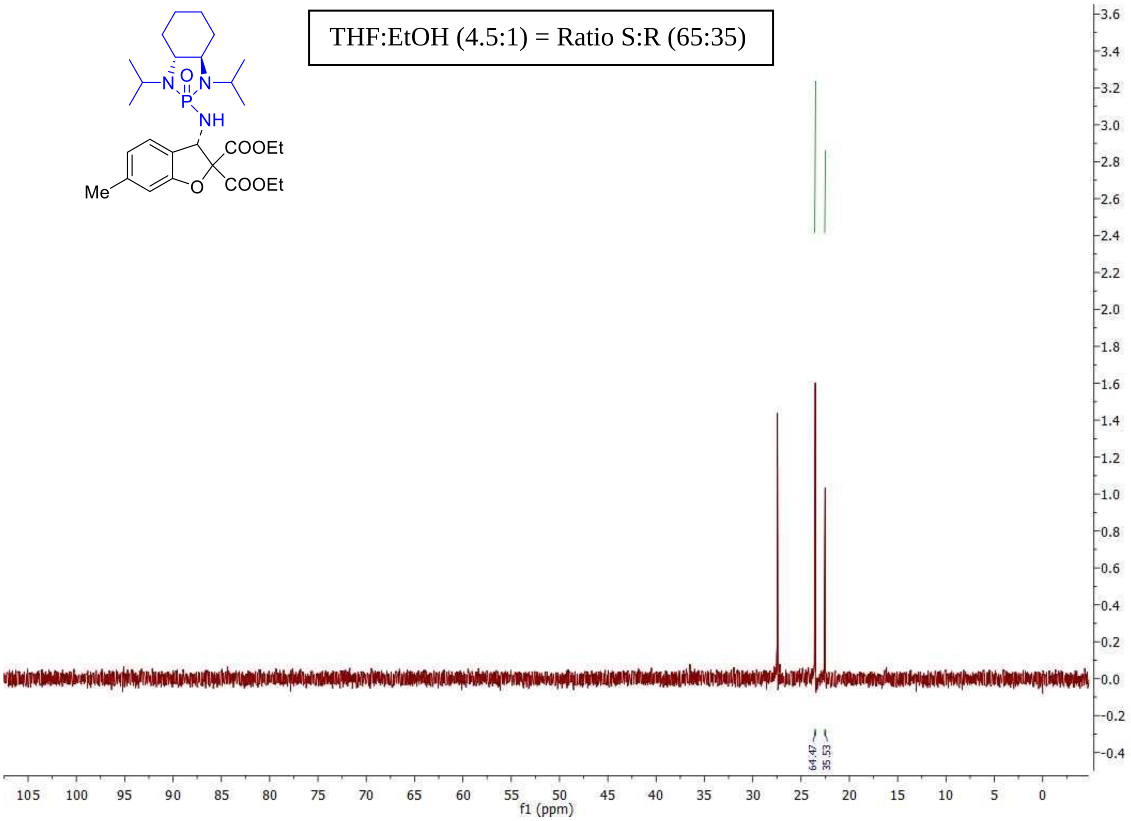
THF:EtOH (4.5:1) = Ratio S:R (65:35)
NH
COOEt
COOEt
Me
64.47
35.53
f1 (ppm)

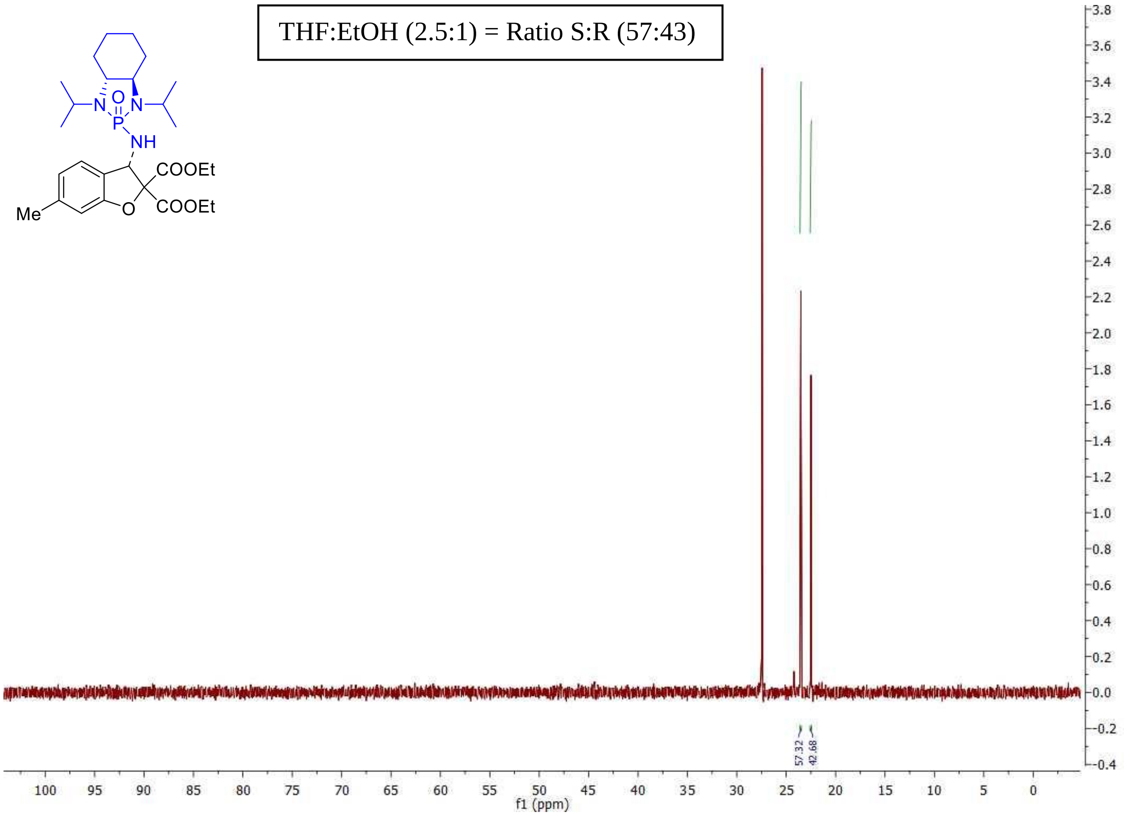
THF:EtOH (2.5:1) = Ratio S:R (57:43)
COOEt
COOEt
NH
Me
57.32
42.68
f1 (ppm)

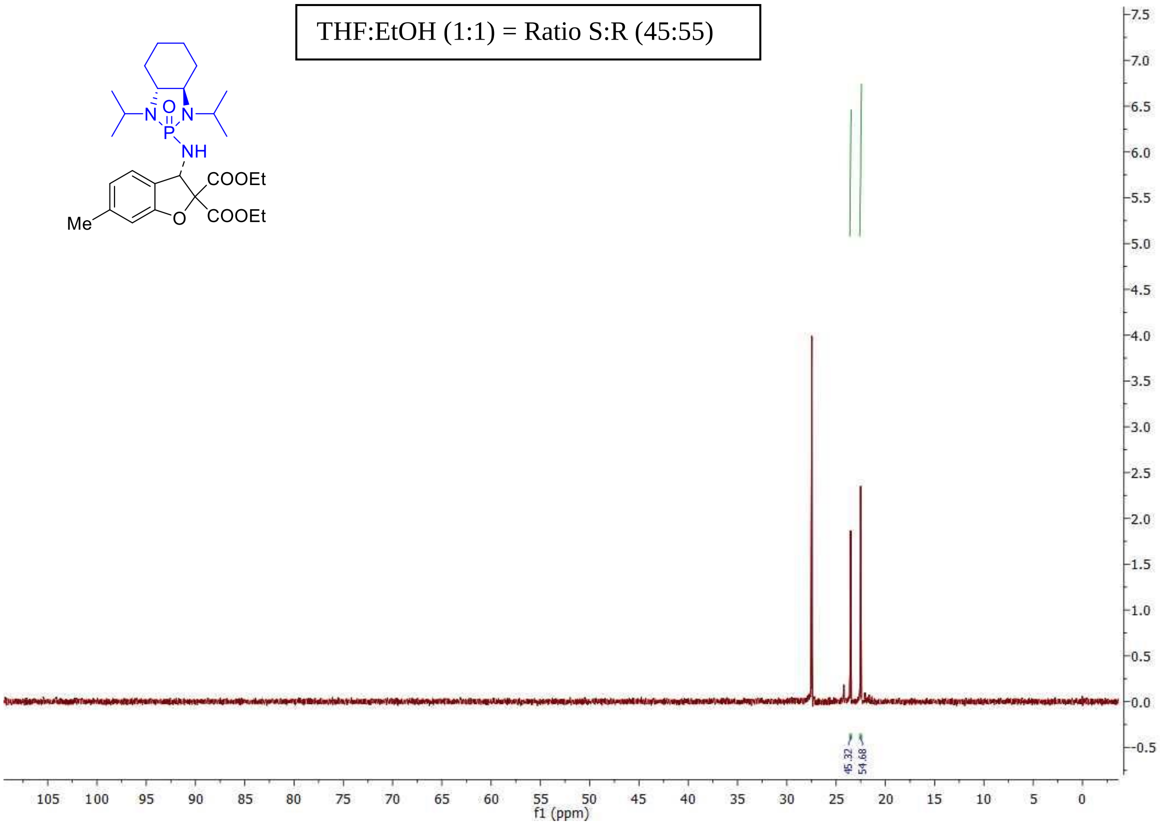
THF:EtOH (1:1) = Ratio S:R (45:55)
COOEt
COOEt
NH
Me
45.32
54.68
f1 (ppm)

THF:EtOH (1:2.5) = Ratio S:R (35:65)

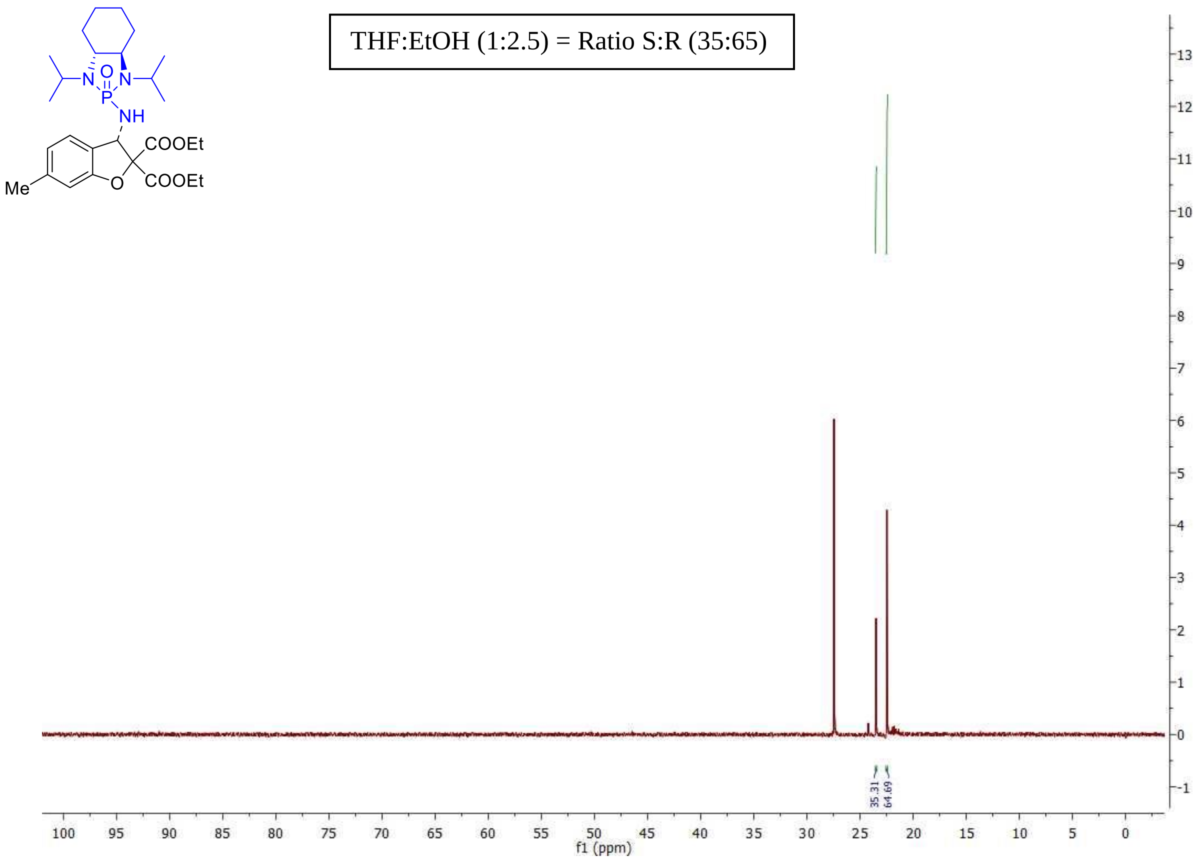

THF:EtOH (1:4.5) = Ratio S:R (31:69)

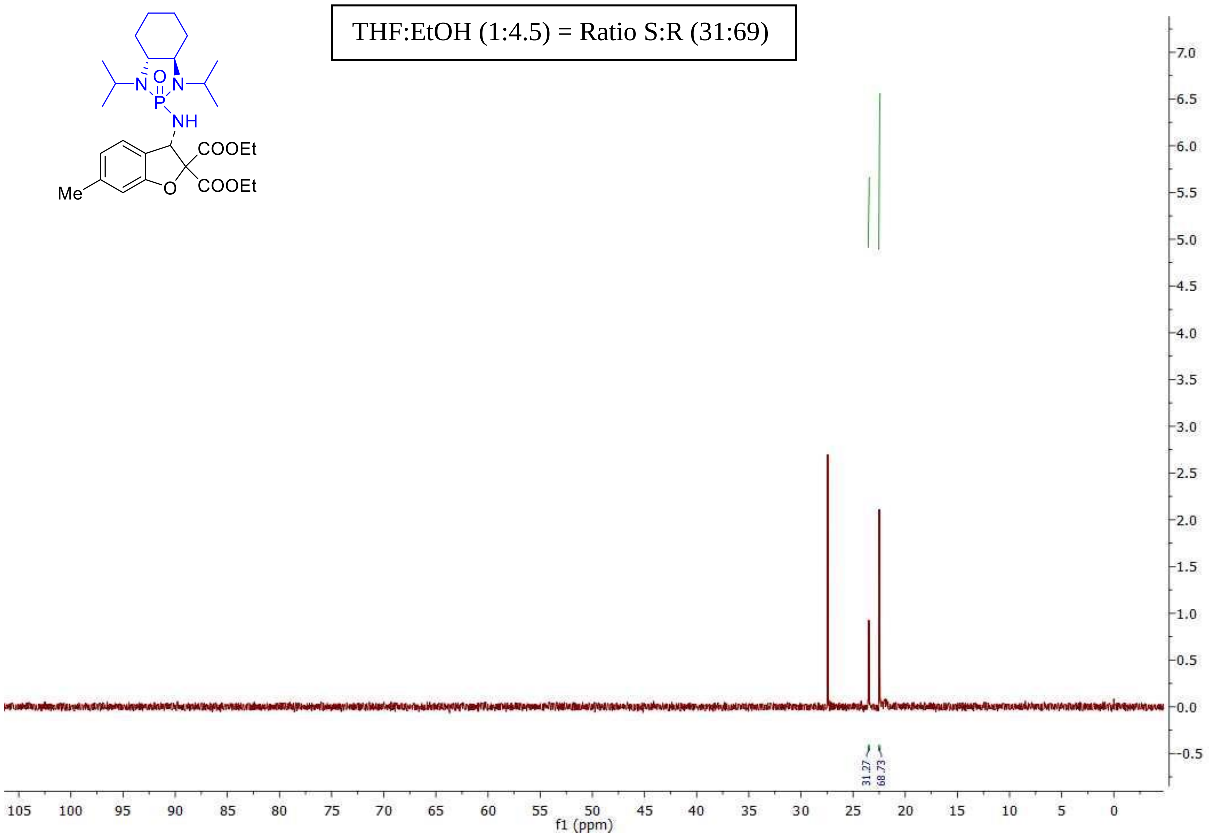

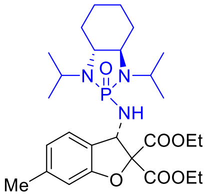

THF:EtOH (1:6.5) = Ratio S:R (30:70)

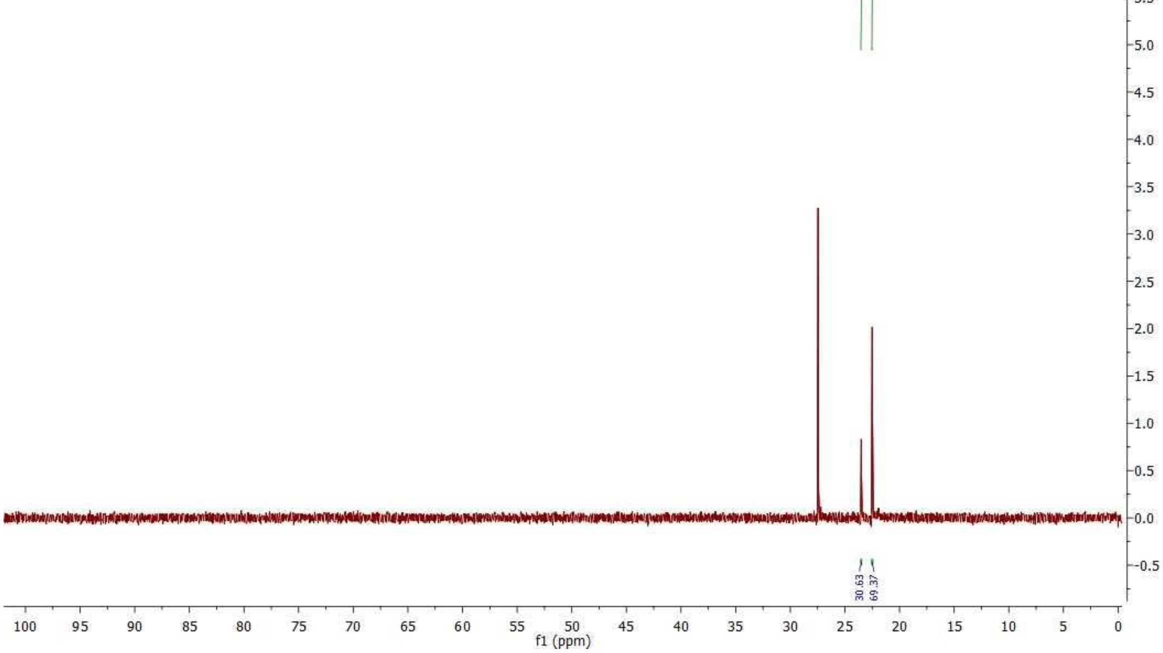

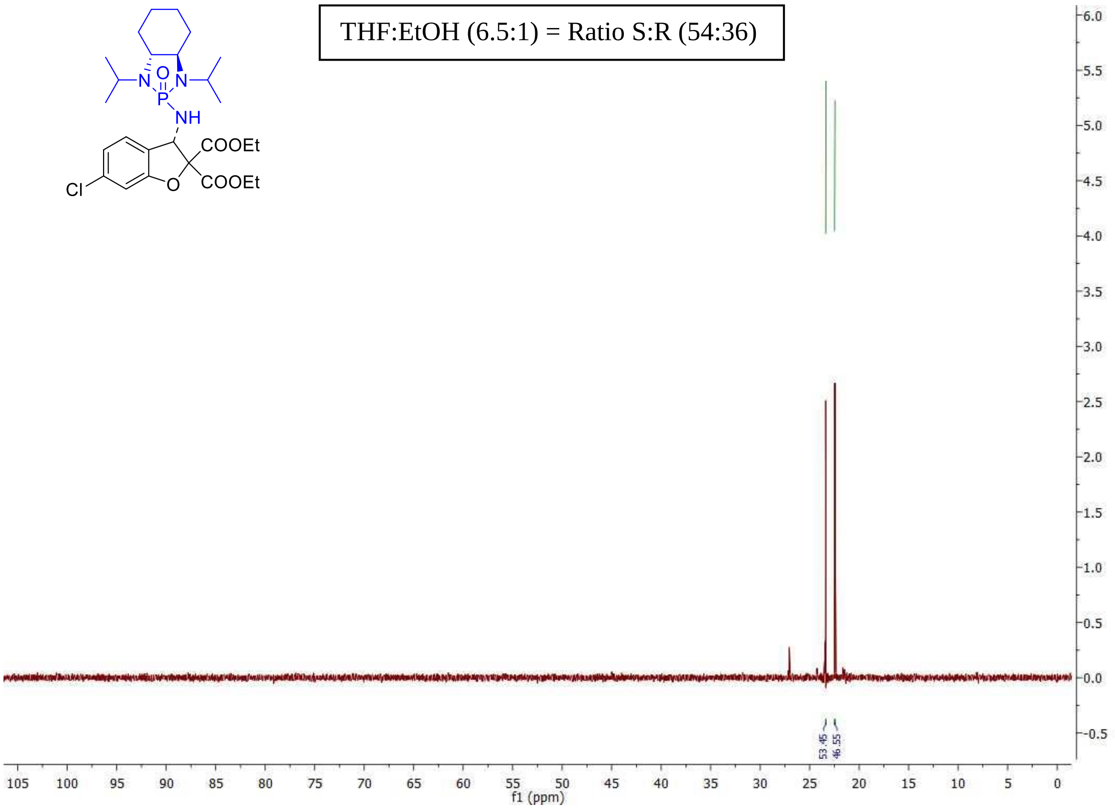
THF:EtOH (6.5:1) = Ratio S:R (54:36)
NH
COOEt
COOEt
Cl
O
N
P
53.45
46.55
f1 (ppm)

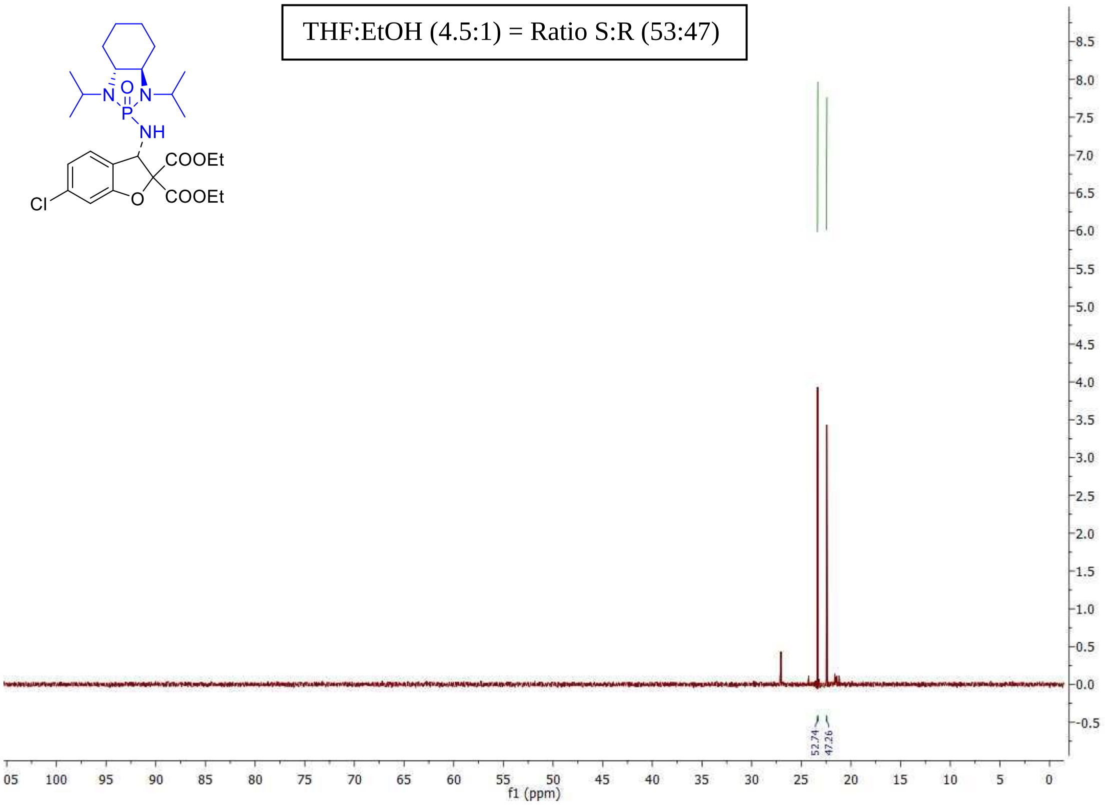
THF:EtOH (4.5:1) = Ratio S:R (53:47)
NH
COOEt
COOEt
Cl
O
N
P
52.74
47.26
f1 (ppm)

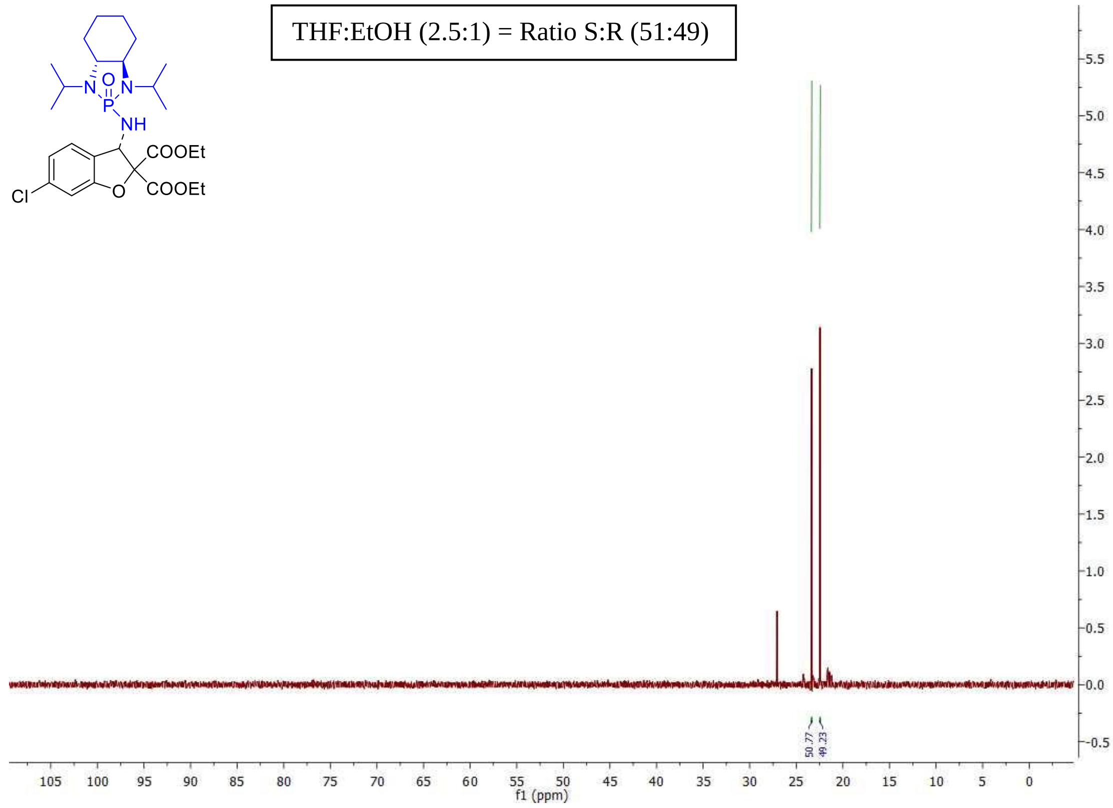
THF:EtOH (2.5:1) = Ratio S:R (51:49)
N
O
P
N
NH
COOEt
COOEt
Cl
O
50.77
49.23
f1 (ppm)

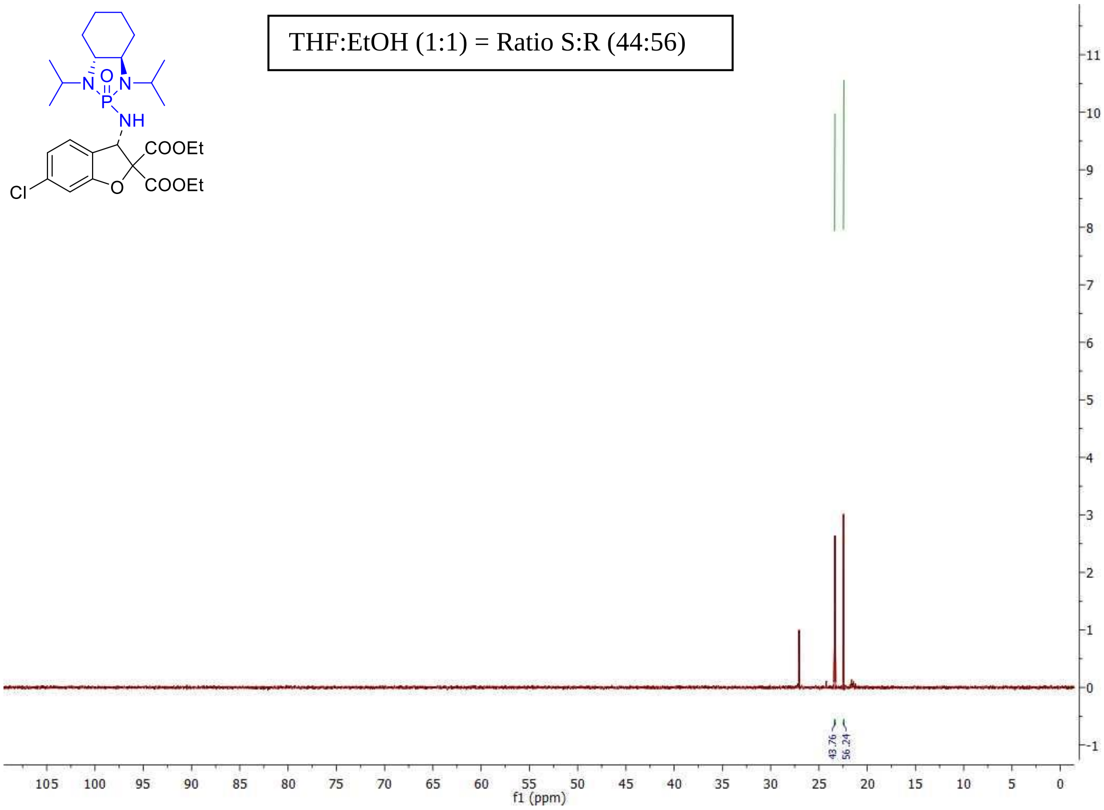
THF:EtOH (1:1) = Ratio S:R (44:56)
N
O
P
N
NH
COOEt
COOEt
Cl
O
43.76
56.24
f1 (ppm)

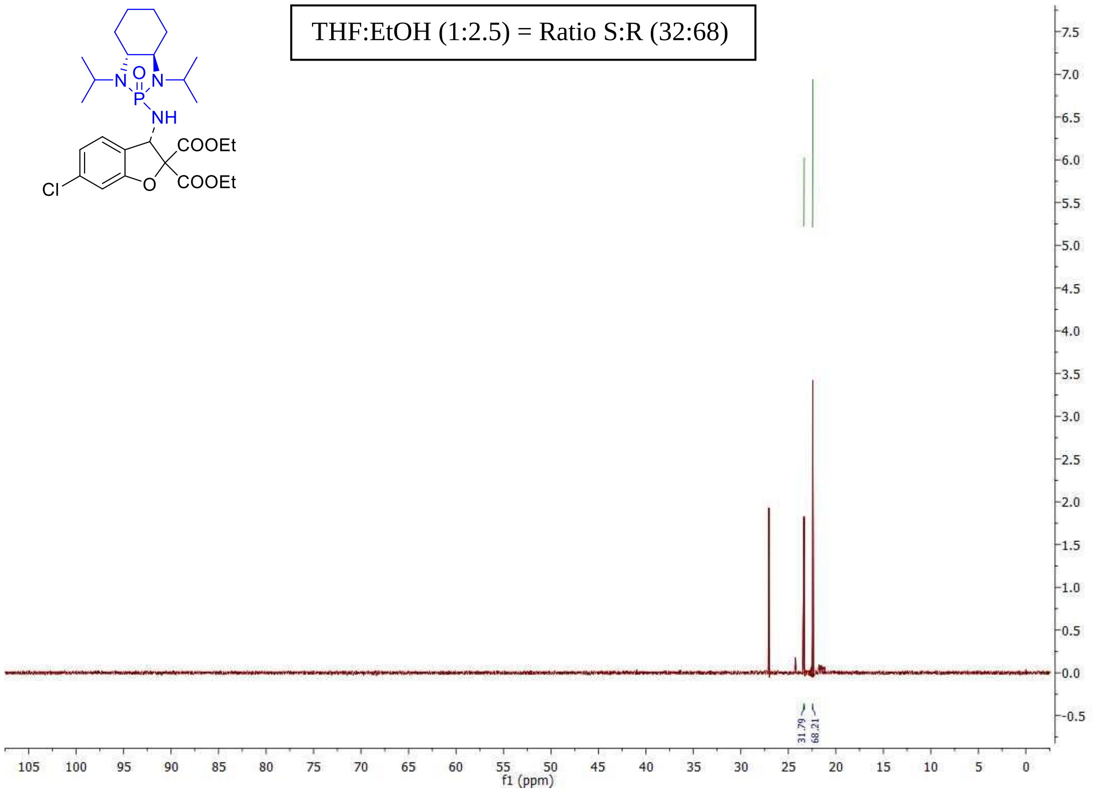
THF:EtOH (1:2.5) = Ratio S:R (32:68)
COOEt
COOEt
NH
Cl
31.79
68.21
f1 (ppm)

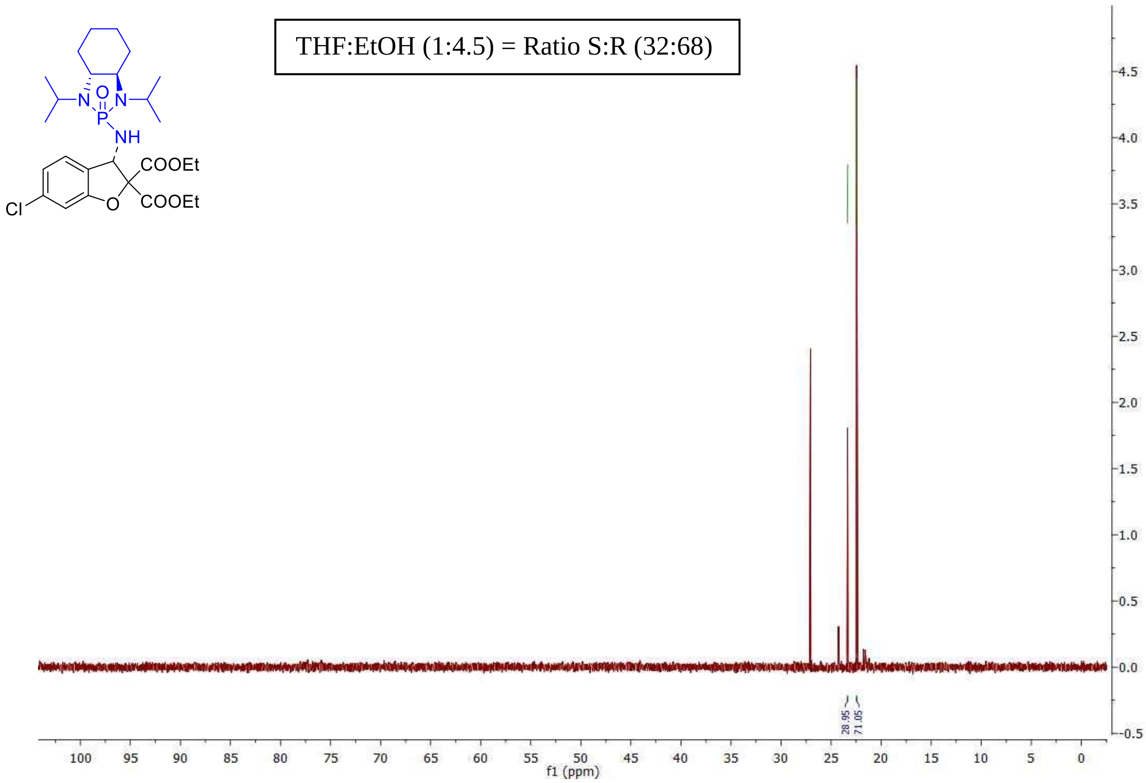
THF:EtOH (1:4.5) = Ratio S:R (32:68)
COOEt
COOEt
NH
Cl
28.95
71.05
f1 (ppm)

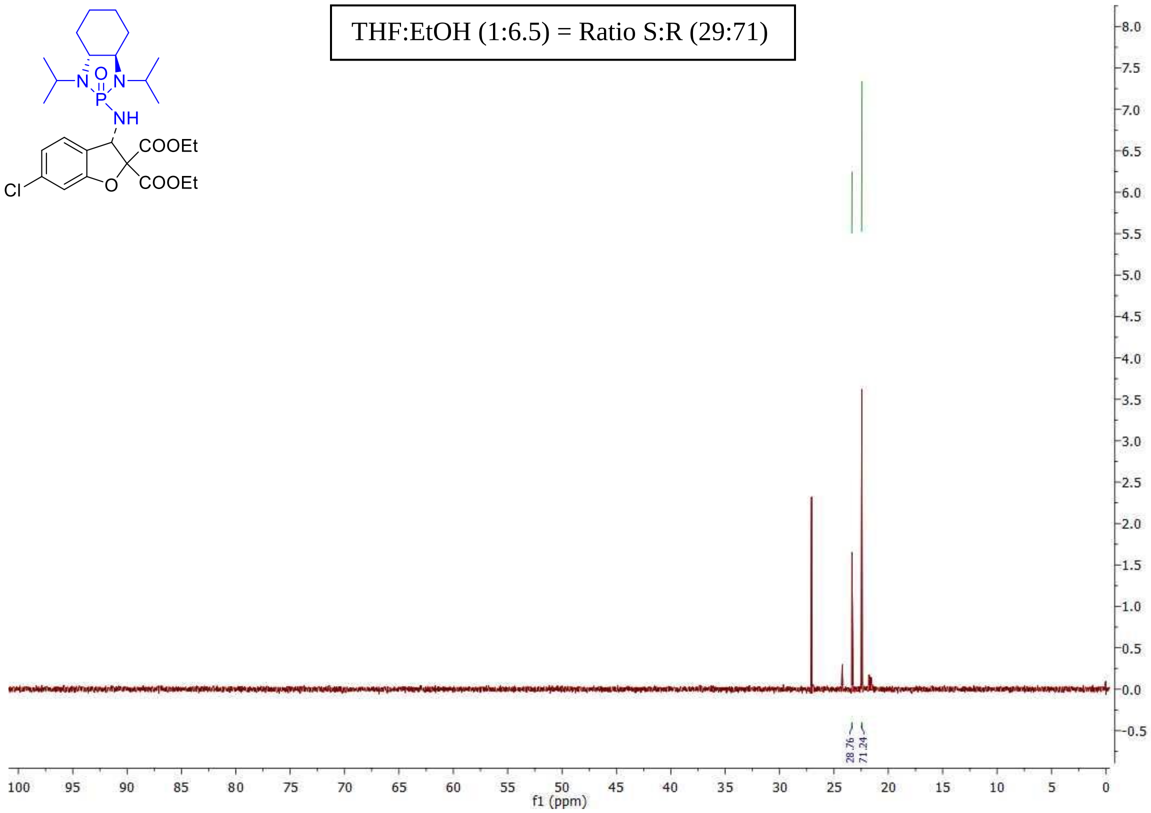

The relationship of dr with optical rotation of mixtures of two diastereoisomers

Compound Structure	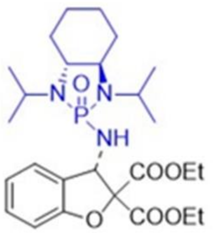	
THF:EtOH	S:R Ratio	$[\alpha]_D^{25}$
6.5:1	67:33	-5
4.5:1	65:35	-6.125
2.5:1	62:38	-9.875
1:1	45:55	-42.375
1:2.5	36:64	-28.5
1:4.5	33:67	-53.625
1:6.5	32:68	-34.5

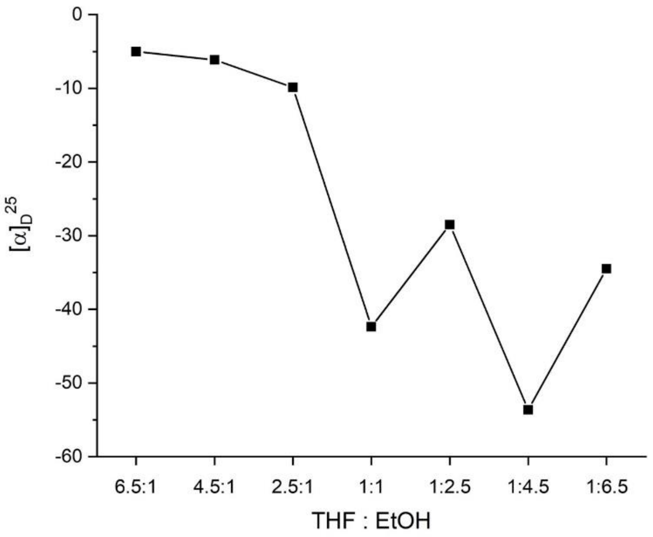

Supplement: Supplementary Materials — General synthesis of salicyl N-phosphonyl imines (1a-1d) and 2,3-dihydrobenzofuran (3a-3e) and their analytical data, 1H NMR spectra, 13C NMR spectra, and 31P NMR spectra. 31P NMR spectra for diastereoselectivity determination. [file 9865108.f1.pdf]
